# Bromelain a Potential Bioactive Compound: A Comprehensive Overview from a Pharmacological Perspective

**DOI:** 10.3390/life11040317

**Published:** 2021-04-06

**Authors:** Arka Jyoti Chakraborty, Saikat Mitra, Trina E. Tallei, Abu Montakim Tareq, Firzan Nainu, Donatella Cicia, Kuldeep Dhama, Talha Bin Emran, Jesus Simal-Gandara, Raffaele Capasso

**Affiliations:** 1 Department of Pharmacy, Faculty of Pharmacy, University of Dhaka, Dhaka 1000, Bangladesh; arkwcky@gmail.com (A.J.C.); saikatmitradu@gmail.com (S.M.); 2 Department of Biology, Faculty of Mathematics and Natural Sciences, Universitas Sam Ratulangi, Manado 95115, North Sulawesi, Indonesia; trina_tallei@unsrat.ac.id; 3 Department of Pharmacy, International Islamic University Chittagong, Chittagong 4318, Bangladesh; montakim0.abu@gmail.com; 4 Faculty of Pharmacy, Hasanuddin University, Makassar 90245, Sulawesi Selatan, Indonesia; firzannainu@unhas.ac.id; 5 Department of Pharmacy, University of Naples Federico II, 80131 Naples, Italy; donatella.cicia@unina.it; 6 Division of Pathology, ICAR-Indian Veterinary Research Institute, Izatnagar, Bareilly 243122, Uttar Pradesh, India; 7 Department of Pharmacy, BGC Trust University Bangladesh, Chittagong 4381, Bangladesh; 8 Nutrition and Bromatology Group, Department of Analytical and Food Chemistry, Faculty of Food Science and Technology, University of Vigo—Ourense Campus, E32004 Ourense, Spain; 9 Department of Agricultural Sciences, University of Naples Federico II, 80055 Naples, Italy

**Keywords:** bromelain, *Ananas comosus*, pineapple, nanoparticles, proteolytic enzymes

## Abstract

Bromelain is an effective chemoresponsive proteolytic enzyme derived from pineapple stems. It contains several thiol endopeptidases and is extracted and purified via several methods. It is most commonly used as an anti-inflammatory agent, though scientists have also discovered its potential as an anticancer and antimicrobial agent. It has been reported as having positive effects on the respiratory, digestive, and circulatory systems, and potentially on the immune system. It is a natural remedy for easing arthritis symptoms, including joint pain and stiffness. This review details bromelain’s varied uses in healthcare, its low toxicity, and its relationship to nanoparticles. The door of infinite possibilities will be opened up if further extensive research is carried out on this pineapple-derived enzyme.

## 1. Introduction

Bromelain is a proteolytic enzyme that contains one sulfhydryl group and is derived from pineapple stems as its most typical industrial source [1], though it is found in all parts of the pineapple. Bromelain has a strong therapeutic potency with a wealth of proteinase inhibitors [2,3]. It is prepared from the juice of cooled pineapple through ultrafiltration, centrifugation, and lyophilization, which results in the production of a yellow powder. This process can succeed in a wide range of pH values. Many chemical agents—including Na_2_S, H_2_S, sodium cyanide, CaCl_2_, and others—facilitate the action of bromelain by acting as stimulatory agents; however, a few of these agents can obstruct proteolytic activity, including Ag+, Hg^2+^, Cu^2+^, and iodoacetate [4]. The process of bromelain extraction and purification is favored in several respects [5], such as in the aqueous two-phase system, reverse micellar system, and chromatographic techniques.

Bromelain elicits an anti-inflammatory response by reducing prostaglandin E2 (PGE-2) and cyclooxygenase-2 (COX-2) synthesis [6]. It also inhibits bacterial enterotoxin production [7], exaggerates the transformation of plasminogen to plasma, interacts with intestinal secretory signaling pathways [8], and retards the MCF-7 cell’s growth-inhibitory response in the mammary epithelium [9]. Bromelain is used to treat osteoarthritis, dental plaque, and gingivitis, and potentiates the therapeutic effects of some antibiotics, e.g., amoxicillin and tetracycline. It is licensed as a complementary therapeutic agent for sinus and nasal swelling and seems to be an important mucolytic agent for rhinitis, rhinosinusitis, and severe rhino-nausea. Bromelain has been widely used as an analgesic agent to treat muscular, arthritic, and perineal pain, as well as pain from an episiotomy. Studies reported that it improves patient quality of life and reduces pain after mandibular third molar surgery. Bromelain can be used to reduce the risk of fatality in those suffering from Peyronie’s disease, and it has been proven effective in treating gastric ulcers in animals [5]. Bromelain can be effective in cancer treatment; it cleaves a cluster of differentiation 44 (CD44) molecules preferentially, thereby inhibiting the first steps of the metastatic process. It has been demonstrated that bromelain has antidiarrheal effects and is purported to be an important nutraceutical treatment for diarrhea [10].

Nanoparticles have a wide range of surface area and pore volume, providing remarkable advantages for absorption and loading of drugs inside pores. Bromelain is a water-soluble polymer that can be chemically changed via free carboxyl groups. The application of nanoparticles in pharmaceutics provides a substantial contribution to modern medical treatment [11]. The most common side effects experienced by people who overdose on bromelain are nausea, vomiting, diarrhea, palpitation, indigestion, loss of appetite, headache, muscle pain, dizziness, drowsiness, and lethargy. Uterine bleeding and heavy menstruation can occur as well [12,13,14].

Bromelain can increase the absorption of medications, including antibiotics, such as amoxicillin and tetracycline; chemotherapy drugs, such as 5-fluorouracil and vincristine; and blood pressure medication, specifically ACE inhibitors, such as captopril (Capoten) and lisinopril (Zestril) [12,15]. Several clinical studies have indicated the therapeutic efficacy and low toxicity of bromelain. In a study conducted by White et al. (1988), rats were given L251-bromelain orally, and blood samples were collected one hour later; the plasma from which was found to contain 0.003% of the administered dose per milliliter [15]. After daily administration of bromelain of up to 750 mg/kg in dogs, no toxicity was found after six months. The lethal dose (LD_50_) of bromelain is greater than 10 g/kg in mice. No carcinogenic or teratogenic effects were observed in rats with dosages of 1500 [10]. However, researchers should continue to study the mechanism of bromelain so that health practitioners can take advantage of its multiaction properties. This review aims to provide a cogent representation of bromelain’s therapeutic efficacy and low toxicity and examine the value of this enzyme in pharmaceutical science.

## 2. Source of Bromelain

Bromelain extract is a fusion of sulfur-containing enzymes, and protein-digesting enzymes known as proteolytic enzymes or proteases. Bromelain also contains other elements in smaller portions. The two primary forms are stem bromelain (EC 3.4.22.32) from the inedible stem and fruit bromelain (EC 3.4.22.33) from the pineapple fruit.

Bromelain can be found in all portions of the pineapple plant (*Ananas comosus*), but the stem is the most typical industrial source, due to its accessibility after harvesting the fruit. Pineapples are traditionally used medicinally in South and Central America. Extra bromelain cannot be obtained through consuming more pineapples as the inedible stem contains a significant portion of the overall bromelain [1]. Bromelain can be obtained from pineapple juice by ultrafiltration [16], but fruit bromelain is commercially unavailable as its properties are different from those of stem bromelain [17]. Commercial bromelain is usually derived from pineapple stems through ultrafiltration, centrifugation, lyophilization [18], and two-step Fast Protein Liquid Chromatography (FPLC) [19]. After extraction, the crude compound containing the bromelain enzyme is purified, as impurities can react with bromelain and obstruct its mechanism of action [20]. Different nonproteases like phosphatases, glucosidases, peroxidases, cellulases, glycoproteins can hinder the mechanism of action of bromelain [21]. For high-level production of recombinant proteins, *Escherichia coli* (*E. coli*) has been used [22] because it is readily available and its genetics thoroughly understood [23]. BL21-AI clone, under separate cultivation states, provided bromelain activity of 9.2 U/mg [24].

## 3. Chemistry and Biochemical Properties of Bromelain

Bromelain is a thiol endopeptidase enzyme combined with other components, including phosphatase, peroxidase, glycoprotein, and carbohydrate. It is obtained from the stem and inchoate fruits of the pineapple through extraction procedures. The result is an aqueous and crude extract belonging to the Bromeliaceae family [8,17,25]. Bromelain undoubtedly has a strong therapeutic potency and possesses many proteinase inhibitors [2,3]. Bromelain from the juice of cooled pineapple undergoes various processes, including ultrafiltration, centrifugation, and lyophilization, which results in the production of a yellow powder. The proteolytic activity of bromelain is conducted by a group of substrates, including gelatin, chromogenic tripeptides, and casein [26,27], and results in several amino acids—tyrosine, alanine, and lysine [28]—from the digestion of proteins. Bromelain has also been found to be useful in the hydrolysis of mackerel [29]. The optimum pH range for bromelain is 5.5–8 [17], but it can work within a wide range of pH values. There are many chemical agents, such as Na_2_S, H_2_S, sodium cyanide, CaCl_2_, that facilitate the action of bromelain by acting as stimulatory agents. Conversely, a few can obstruct bromelain’s proteolytic activity, including Ag^+^, Hg^2+^, Cu^2+^, and iodoacetate [4].

## 4. Biosynthesis of Bromelain

**Figure 1:**
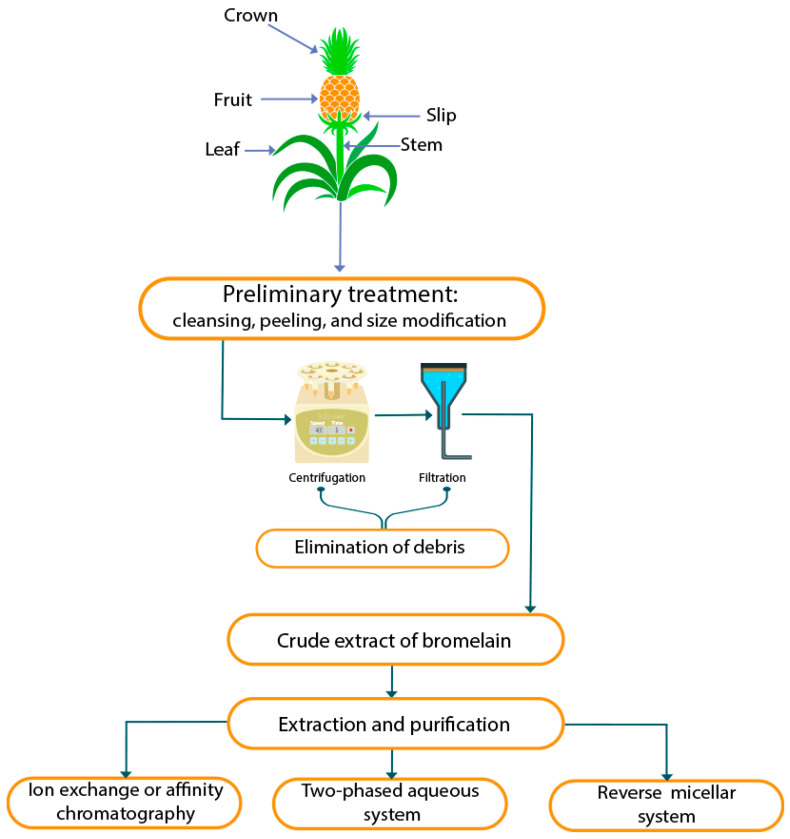
Overview of extraction and purification of bromelain.

Bromelain can be collected in several ways from pineapple plants, which are valuable for their market potential, as well as for their therapeutic applications. The bromelain known as stem bromelain is commercially available, and it is therapeutically better than fruit bromelain. The isolation of bromelain can be performed to produce either stem or fruit bromelain based on the desired therapeutic activity [25]. The process of bromelain extraction and purification has several steps [5], as delineated below (Figure 1).

### 4.1. Aqueous Two-Phase System (ATPS)

A useful and cost-effective approach for extracting and purifying protein and enzyme mixtures is the aqueous two-phase technique. The ATPS consists of either two different polymers—polyethylene glycol (PEG) and dextran—or one polymer and one salt—PEG and a phosphate salt. The two polymers separate into phases when phase-forming substances undergo solubilization in aqueous media upon the critical concentration. Unwanted byproducts—e.g., proteins, pigments, and polysaccharides that can reduce enzymatic action—may be eliminated through this method [30,31]. The bromelain component is preferentially partitioned in the PEG phase and polyphenol oxidase in the potassium phosphate phase. The partition coefficient tends to decrease with the higher molecular weight of polyethylene glycol [30]. In ATPS, high active enzyme recovery is attributable to PEG’s modification of the enzyme’s active sites. Ketnawa et al. (2010) found that ATPS for the isolation of bromelain from pineapple bark produces 206% and 113.54% successful recovery with 3.44- and 2.23-fold purification, respectively [5,31].

### 4.2. Reverse Micellar System

Reverse micellar extraction (RME) is an effective liquid extraction strategy with a thermodynamically safe approach for downstream processing of biomolecules [32]. Reverse micelles are surfactant-stabilized droplets of water that spread in organic solvents [33]. Surfactants, commonly referred to as amphiphiles, are generally organic compounds containing both hydrophilic and lipophilic regions [34]. Some of the benefits of RME include a substantial amount of original activity, simplicity of scale, and the ability for continuous service [32,35]. In this method, the purified bromelain is contained in the micelle, and impurities remain in the organic aqueous layer. Furthermore, using a cationic surfactant (butanol, cetyltrimethylammonium bromide, or hexanol) for RME can obtain 95.8% recovery and 5.9-fold purification. Ultrafiltration methods further result in 8.9-fold purification [5,36].

### 4.3. Chromatography Technique

Chromatography methods are commonly used to purify bromelain [5] and preserve the refined protein structure. Many conventional methods are used in bromelain purification, including gel filtration chromatography, ion-exchange chromatography, affinity chromatography, and high-speed countercurrent chromatography (HSCCC), among others [37,38,39]. The isolation and purification of the protein in solution (mobile phase) are performed going to the contact of the stationary phase [40]. Typically, liquid chromatography (LC) purification materials are costly, due to the expense of the ingredients used in the manufacturing phase [41]. Although many techniques exist, one of the most relevant strategies is ion-exchange chromatography. It is a highly precise, versatile, efficient, and cheap purification technique. Cationic-exchange chromatography results in 10-fold purification of bromelain [42]. According to Yin et al. (2011), HSCCC can result in 3.01 g purified bromelain from 5 g crude extract of bromelain [43]. An immobilized metal affinity membrane (IMAM) was used for bromelain purification, by which 15.4-fold purification and 94.6% recovery were achieved [5].

## 5. Bioavailability of Bromelain

In a study by White et al. (1988), rats were given L251-bromelain orally, and blood samples were collected at certain intervals. The I-bromelain stock was diluted in normal saline. Each animal was administered a 1.0 mL bromelain solution, which contained 0.87 mg I protein. Each animal was administered 2–21 µCi of Na 125I (equivalent to twice the level of free I in the 12SI-bromelain sample) diluted in normal saline. A maximum level of 270 ng/mL bromelain was found one hour after administration. Ten percent of the trichloroacetic acid could precipitate approximately 40% of the I protein in plasma. One prominent peak of radioactivity was shown in the plasma samples by the electrophoretic analysis, with a molecular weight of 26–32,000 Daltons. The L251 compounds that were TCA precipitable, the total radioactivity, and the I protein’s molecular weight profile in the plasma were determined. This peak showed 0.003% of the administered dose per milliliter contained in the one-hour plasma sample [15].

## 6. Therapeutic Efficacy of Bromelain

Bromelain in pineapple is a type of enzyme known as a protease, which breaks other proteins apart by cutting the chains of amino acids. Even more specifically, bromelain is a cysteine protease, meaning that it breaks apart proteins wherever they have a cysteine amino acid [21]. Bromelain, the pineapple protease, selectively prevents proinflammatory prostaglandins’ biosynthesis obviously via indirect intervention. Endogenous proteases which accompany trauma or repeated exposure to too much stress significantly raise the relative proportions of those prostaglandins which have inflammatory symptoms. The sensitivity of bromelain has been shown to be similar to the endogenous protease plasmin. Bromelain works on fibrinogen to have drugs similar to plasmin products, at least in effect. Small molecular weight active peptides are used to control prostaglandin biosynthesis and to establish conditions in a stable body. Significant amounts of orally ingested bromelain have been found to be absorbed into the bloodstream unchanged, thereby increasing the proteolytic and fibrinolytic blood activity for hours. The comparison of benefits from the aspirin-like medications with bromelain, though bromelain does not induce any of the other’s unwanted side effects, shows bromelain is distinct from those from nonsteroidal anti-inflammatory medicines to the prostaglandin synthetic pathway. Although aspirin inhibits cyclooxygenase, and hence, the biosynthesis of all prostaglandins, the arachidonate cascade at the thromboxane synthetase level is assumed to be further inflamed with bromelain. Circumstantial evidence indicates the synthesis of the “proinflammatory” prostaglandins inhibited with bromelain without influencing the “anti-inflammatory” prostaglandins. Therefore, bromelain helps to restore the equilibrium of the two prostaglandins, which define the condition of the healthy organisms [44]. Though bromelain can be used in a wide range of areas (discussed in Table 1), the most traditional and established use of it is as an anti-inflammatory agent. Inflammation is pivotal in the development of cancer during cellular transformation, angiogenesis, proliferation, metastasis, and invasion. It has been demonstrated that suppression of chronic inflammation may reduce cancer incidence and also inhibit cancer progression. Bromelain, a bioactive enzyme, can serve the purpose at a comparatively low cost. That is why it is very useful in medical sciences [45].

### 6.1. Anticancer Activity

Studies have shown that bromelain has anticancer effects [9,46,47]. While bodies usually regulate cell proliferation and growth, cell-cycle disparities can lead to the corruption of cell growth and transform a normal cell into a cancerous one. Several mechanisms inside cells protect their DNA from harmful genomic variability and toxins [9]. However, when cells lose checkpoint controls, they are subject to unusual regulation of the cell cycle and can ultimately become tumorous [46]. One study demonstrated that bromelain acts in rat tumor cell lines and inhibits cell growth [48]; specifically, the advancement of Kato-III cell lines in gastric carcinoma significantly decreased with bromelain therapy. Bromelain retards MCF-7 cell growth inhibitory response in the mammary carcinoma cells and stimulates the autophagy cycle. Monocytic cytotoxicity in breast cancer is promoted by bromelain via oral administration [47]. 5-fluorouracil (5-FU) is a well-known drug in cancer treatment. It is used to treat breast, colon, stomach, rectal, and pancreas cancer. Uses of bromelain in cancer treatment are discussed below:

#### 6.1.1. Breast Cancer

Bromelain is a potent antitumor agent [49] that inhibits the growth of MCF-7 cells in mammary carcinoma, initiates the autophagy process, and induces cancer cell death (apoptosis) [50]. It also promotes monocytic cytotoxicity in breast cancer patients when taken orally [47]. An increased dosage of bromelain facilitates apoptosis of breast cancer cells in particular [51].

#### 6.1.2. Melanoma and Epidermoid Carcinoma

Bromelain was found to have effective anticancer action against cell lines of melanoma and epidermoid carcinoma. Bromelain not only minimized their proliferation, but also decreased COX-2 gene expression [52], caused apoptosis, and suppressed melanoma cell metastasis [5]. Bromelain has effectively decreased the amount of CD44—a surface protein found in human Molt 4/8 leukemia cells [53].

#### 6.1.3. Colorectal Cancer

Colon cancer (CC) and rectal cancer (RC) are often referred to in combination as colorectal cancer (CRC) [54]. Bromelain’s pathways have not been thoroughly studied in CRC. However, studies on CRC in zebrafish and xenograft mice found that bromelain suppressed cell line growth and tumor formation. Thus, bromelain may provide a better solution for CRC than present therapies. Bromelain also caused high levels of ROS and superoxide, plus autophagosome and lysosome formation. High rates of apoptosis and elevated concentrations of apoptotic proteins, such as Endo G and caspase-3,-8, and -9 were also induced in the qPCR study [55].

#### 6.1.4. Pancreatic and Hepatic Cancer

Due to its thick extracellular matrix (ECM), pancreatic cancer is one of the most difficult cancers to treat. Bromelain is said to degrade ECM in cancerous tissue, but its half-life in the blood is short, and therefore, has low tissue aggregation. Researchers have developed a reversible technology for PEG-modification that can sustain protein levels in the blood, while preserving their function. SPRA-bromelains are highly active, have longer retention in the blood, and have a high aggregation of tumors. It could be an effective drug delivery mechanism for pancreatic cancer [56]. Current systemic dosages of chemotherapeutic medications, such as gemcitabine, 5-FU, cisplatin, and doxorubicin, are given every seven days over four cycles, due to systemic toxicity. The combination of bromelain and N-acetylcysteine demonstrated synergy in both pancreatic and hepatic tumor cell lines. A CI value of <0.5 implied that it was possible to significantly decrease the existing clinical chemotherapeutic dosage with concurrent use of bromelain. Synergistic combinations resulting in significant dose reduction of chemotherapy can allow higher efficacy treatment to be more frequent [57].

### 6.2. Anti-Inflammatory Effect

**Figure 2:**
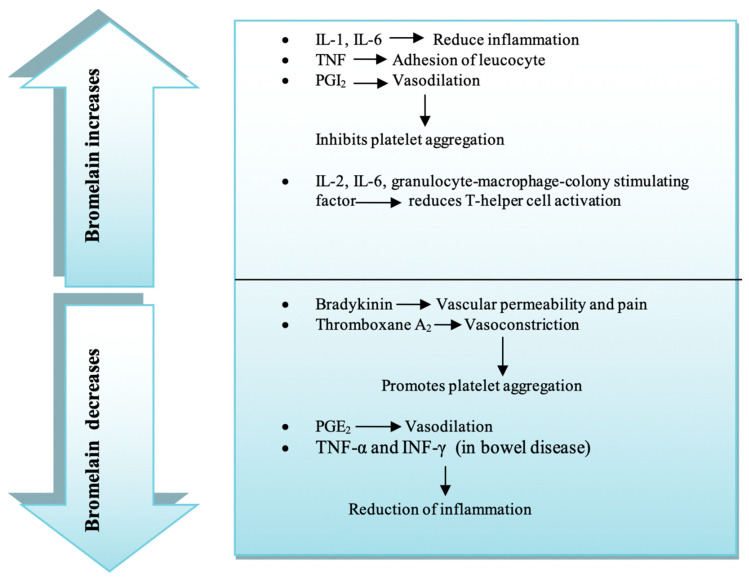
Effect of bromelain in acute inflammation.

Inflammation is considered to be a complex biological mechanism that is primarily regulated by tissue homeostasis disruption [58]. Inflammation is important for cancer development, specifically in the cellular transmutation, reproduction, angiogenesis, invasion, and metastasis stages. Suppressing chronic inflammation can, therefore, reduce the incidence of cancer, and cancer progression may be inhibited [59]. COX-2, which is engaged in the synthesis of PGE-2, is an indispensable element of cancer-associated inflammation. As a proinflammatory lipid, PGE-2 exhibits an immunosuppressing effect and promotes the progression of the tumor. COX-2 converts Arachidonic acid into PGE-2, favors tumor angiogenesis, and elevates the risk of cancer progression [60]. COX-2 and PGE-2 expression levels in murine microglial cells and individual monocytic leukemia cell lines are downregulated by bromelain [61]. Bromelain stimulates Interleukin (IL)-1β, tumor necrosis factor (TNF)-α, IL-6, and interferon (INF)-γ, which are regarded as the inflammatory mediators in human peripheral blood mononuclear cells (PBMC) and mouse macrophages [62,63,64]. Two decisive mediators of the immune response, substance P and PGE-2, had lower concentrations in mice treated with bromelain. As a result, inflammatory exudates were lower in the mouse model of acute knee joint inflammation [60]. After being treated with bromelain, two ulcerative colitis patients, 60 and 67 years of age, respectively, recovered from their condition. Endoscopic testing in both cases validated the progression from the initial state [65]. These results suggest that a healthy immune system could potentially be achieved, along with the prompt response to cellular pressure, by using bromelain. On the other hand, bromelain decreases IL-1β, TNF-α, and IL-6 excretion when immune cells are already stimulated during inflammation-induced excess production of cytokines [66,67]. Scientists have found that bromelain decreases the expression of TNF-α and INF-γ in bowel disorders that cause inflammation [68]. Immune cells and cancer cells both express CD44 (the cell surface marker), which is required in the growth of cancer cells and metastasis. Moreover, CD44 arranges adequate lymphocytes at the inflammatory site [69,70]. Transforming growth factor (TGF)-β is one of the vital regulators of inflammation. Bromelain modulates its expression in rheumatoid arthritis and osteomyelofibrosis affected patients [71,72]. Bromelain activates NK cells and increases the generation of IL-2, IL-6, and granulocyte-macrophage colony-stimulating factors. It also reduces T-helper cell activation [73,74]. In rats with subcutaneous inflammation caused by carrageenin, the effect of bromelain was investigated. Bromelain caused substantial decreases in both PGE2 and substance P in the exudate after oral in vivo administration (10 and 20 mg/kg p.o) [60]. The thermal hyperalgesia and allodynic mechanical indices of neuropathic pain were greatly reduced by bromelain. In rats treated with bromelain, there have been changes in the sciatic and structural integrity. These rats demonstrated considerable changes in nuclear transcription factors of the sciatic nerve [75]. In this way, the majority of inflammatory mediators are reduced by bromelain, though it is crucial to anti-inflammatory treatment in different situations (Figure 2) [76].

### 6.3. Antimicrobial Effect

Bromelain acts as an antibacterial agent by inhibiting the growth of intestinal bacteria, such as *Vibrio cholera* and *Escherichia coli* (*E. coli*). Bromelain stops enterotoxin production of *E. coli* (ETEC) bacteria and prevents diarrhea caused by *E. coli*. ETEC infection could be eradicated using bromelain as prophylaxis [7]. Bromelain can be utilized as an anthelmintic agent against gastrointestinal nematodes like *Heligmosomoides polygyrus*, *Trichoderma viride*, and *Trichurismuris* [77]. The synergistic impact of bromelain has also been observed when used concurrently with antibiotics. It is, therefore, evident that it can be used to destroy distinct intestinal pathogenic organisms. Bromelain can treat fungal infections as well [78]. Pityriasis lichenoides chronica is a skin disorder that produces tiny, scaling, raised spots on the skin [79], and bromelain can effectively heal it [80].

### 6.4. Effect on Blood Coagulation and Fibrinolysis

Fibrinolysis is the enzymatic breakdown of fibrin in blood clots and secure clearance of clot fragments [81]. Bromelain effectively performs fibrinolysis and restricts coagulation of blood [82]. It exaggerates the transformation of plasminogen to plasma, which in turn hinders the synthesis of fibrin (a protein required in the coagulation of blood) [8]. The concentration of fibrinogen in serum is also reduced by bromelain. By suppressing ADP-induced aggregation of platelets, bromelain delays both prothrombin time (PT) and activated partial thromboplastin time (APTT) [83,84]. Both intrinsic and extrinsic pathways result in fibrin formation. However, bromelain limits its formation by reducing some of the intermediates of clotting cascades (specifically, factor X and prothrombin) and increasing fibrinolysis. It also reduces prekallikrein (PK), and thus, inhibits the generation of bradykinin at the site of inflammation. As a result, it reduces edema and pain, while increasing circulation at the injury site [85].

### 6.5. Antiplaque Effect

Dental caries are prevented by brushing teeth more frequently, thereby lessening the duration of tooth contact with leftover food particles. Antiplaque agents in toothpaste help prevent decay as well [86]. As reported by Harmely et al. (2011), 5% stem bromelain is beneficial in toothpaste as an antiplaque agent [87]. Rahmadini (2013) carried out a similar research formula in sampling rough bromelain from the hump of pineapple in toothpaste and examining their mechanical resistance for 28 days [86].

### 6.6. Effect on Chronic Wounds

Bromelain is thought to be beneficial for soft tissue wound healing, due to escharase, one of its components. Howat and Lewis carried out a double-blind and controlled clinical experiment reviewing the impacts of bromelain on episiotomy injuries. As a result, they claimed a quicker reduction rate of edema and contusion in subjects who took bromelain compared with cases where a placebo was used [88,89,90]. Severe, full-thickness wounds are healed more quickly with timely debridement and removal of eschar to decrease wound bioburden [84,85]. For burn wounds, effective eradication of the eschar within 72 h is recommended [90]. Bromelain used as a cream contains 35% bromelain in a lipid base and assists in necrotic tissue debridement, hastening recovery, due to the presence of escharase [90,91]. Natural protein substrates and some glycosaminoglycan substrates cannot be hydrolyzed by it [17]. In case of postoperative injuries and easing patients’ pain and inflammation, bromelain remains first on scientists’ priority list because bromelain can improve the debridement mechanism and provide quicker healing and more effective re-epithelialization [92].

### 6.7. Treatment of Osteoarthritis

The most prevalent form of arthritis in the United States is osteoarthritis, and its rate in the population varies from 3.2% to 33%, depending on the joint [17,93]. Bromelain is an effective solution for this disease, due to its analgesic effects, which are believed to arise from its direct effect on pain mediators, such as bradykinin [94,95]. Bromelain is being used as a benign substitute, and researchers have identified significant efficacy of bromelain in arthritis. Nonsteroidal anti-inflammatory drugs (NSAIDs) are widely used for arthritis pain, but bromelain could act as a replacement agent that works similarly to NSAIDs [17,96]. A clinical study on 103 knee arthritis patients compared bromelain, rutin, and trypsin treatment to diclofenac, and the result was the same for both agents as a pain reliever [17,97].

### 6.8. Antibiotic Potentiation

Bromelain can enhance tissue permeability and absorption of antibiotics after being administered orally, subcutaneously, or intramuscularly [92,98,99]. In humans, bromelain raises levels of antibiotics in urine and blood. After bromelain is administered, higher blood and tissue levels of amoxicillin and tetracycline can be observed [100]. As a result, higher serum and tissue levels of the drug can be maintained. Thus, bromelain potentiates the efficiency of antibiotics and lessens side effects [101]. Diseases like pyelonephritis, cutaneous Staphylococcus infection, rectal abscesses, sinusitis, cellulitis, bronchitis, pneumonia, and thrombophlebitis can be more quickly treated by using bromelain and antibiotic therapy concurrently. Bromelain vastly increases the efficacy of antibiotics in a variety of conditions [12,102].

### 6.9. Anthelmintic Potential

Helminthiasis is a parasitic worm infection proving increasingly resistant to anthelmintic drugs. Researchers are conducting in vivo studies to develop alternative strategies to protect animals and humans from infection [103], including evaluating the potency of bromelain as an anthelmintic agent. Shukuru et al. 2020 studied the anthelmintic potency of chitosan-encapsulated bromelain in the gastrointestinal tracts of East African goats and determined it to be safe; however, when delivered as a single dose, it showed poor performance against strongyle nematodes of the gastrointestinal tract [104]. Similarly, an in vitro analysis was performed by Rakesh et al. (2016) to evaluate the anthelmintic efficacy of bromelain against goat gastrointestinal nematodes. In this research, the authors assessed the in vitro action of stem bromelain in reducing gastrointestinal nematodes in goats. The anthelmintic behavior of bromelain was analyzed using larval development assay (LDA) and egg hatch assay (EHA) at different concentrations. Bromelain exhibited major adulticidal activity on *Haemonchus contortus* at a concentration of 150 μM, destroying all worms by disrupting their cuticles after 8 h of incubation and eventually inducing worm disintegration [105]. Important adulticidal action on *Haemonchus contortus* to destroy all worms, damage their cuticle after 8 h of incubation, and eventually cause worms to disintegrate was found as anthelmintic activity in bromelain [105].

### 6.10. Immunomodulatory Effect

**Figure 3:**
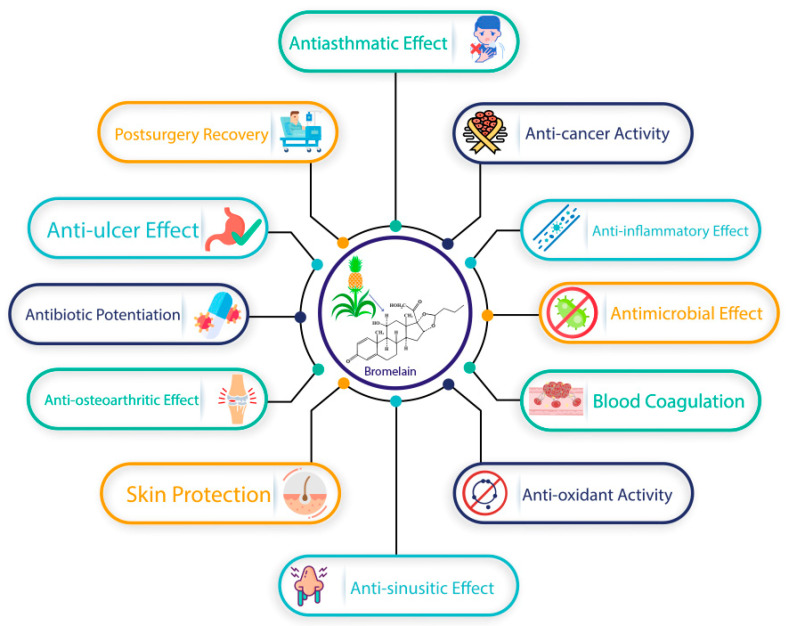
An overview of the significant therapeutic effects of bromelain.

Bromelain has been noted for its immunomodulatory effect in various epidemiological studies as it can both activate and suppress the immune system. It enhances the activation of CD2-mediated T cells. It also improves the antigen-independent attachment of T cells to splenocytes and raises the development of human peripheral blood mononuclear cells in IFN-γ dependent TNF-α, IL-6, and IL-1α. In addition to promoting the action of T cells, bromelain can also prevent T cell responses [63]. Saptarini et al. (2020) conducted an experimental in vivo study to establish the immunomodulatory action of crude bromelain from pineapple. The phagocytic activity was noted in carbon removal from black ink-induced mice, whereas, from antibody hemagglutination to the mouse that challenged sheep red blood cells, the specific humoral immune response was observed. The results show that the immunomodulatory behavior established at 1.56 mg, 3.12 mg, and 4.68 mg/20 g body weight in mice where 3.12 mg/20 g exerts the effect compared to the standard [106]. The overview of the significant therapeutic effects of bromelain is shown in Figure 3.

### 6.11. Treatment of Sinusitis

Bromelain has been proposed as an alternative treatment for sinusitis. Tentative findings indicate that it may minimize congestion, increase ease of breathing, and suppress coughing when administered orally. It is licensed as a therapeutic agent for sinus and nasal swelling and inflammation following operations on the nose, ear, and throat by the German Commission E. An analysis of three minor, but well-designed trials showed that bromelain helps to alleviate symptoms of sinus infections [12].

Rhinosinusitis is marked by acute irritation of the nasal passage and paranasal sinuses, while chronic rhinosinusitis (CRS) triggers mucus membrane destruction and is more severe and long-term. Bromelain could be an important mucolytic agent for allergic rhinitis, rhinosinusitis, and severe rhinosinusitis by decreasing proinflammatory prostaglandin production and reducing swelling in nasal routes [5]. Bromelain also significantly reduces mucus production and improves drainage.

### 6.12. Antinociceptive Effect

Clinical and experimental evidence has shown that bromelain has analgesic properties, and thus, it has been widely used to treat muscular and perineal pain, and pain from arthritis and episiotomy [107,108]. Studies have reported that bromelain improves the quality of life and eases pain after mandibular third molar surgery. Walker et al. (2002) asserted that bromelain is dose-dependent in its effectiveness for alleviating mild knee pain [109]. The antinociceptive effect of bromelain on neuropathic pain from chronic constriction injury (CCI) in Wistar rats showed that hyperalgesia and allodynia were mitigated by bromelain after twenty-one days of treatment [75]. Another study using bromelain to treat CCI showed that bromelain maintained a neuronal electrolyte (Ca^2+^, Cl^−^, Na^+^, and K^+^) imbalance [110]. It has also been reported that bromelain is an antioxidant that stimulates antioxidant enzyme secretion of catalase, superoxide dismutase, and reduced glutathione via increased concentration of nuclear factor (erythroid-derived 2)-like-1 and -2 (NrF-1 and NrF-2) [75]. Nitric oxide synthase expression is mitigated by bromelain, thereby inhibiting nitric oxide and reactive nitrogen species production.

### 6.13. Treatment of Peyronie’s Disease

Peyronie’s disease is characterized by a severe curvature of the erect penis caused by plaque or a hard lump that forms on the appendage and affects more than 1% of males between 45 and 60 years of age. In critical cases, the condition causes tremendous pain during an erection, making it impossible to take part in sexual activity. There is no cure, and although there are three types of surgery available, no surgical options have proven to be consistently effective. Instead, surgery increases the risk of impotence or further penile deformation. The cause of Peyronie’s disease is unknown, but it occurs in the older population, possibly due to aging-related enzyme depletion, similar to age-related eyesight, hearing, and memory loss. A buildup of scar tissue on the penis can lead to Peyronie’s disease [61]. If the plaque, collagen, or other components of the deformity make their way into the bloodstream, there are inadequate enzymes to break up the foreign substances, making the disease potentially fatal. Bromelain has the potential as a remedy for Peyronie’s disease by breaking up collagen in the bloodstream, thereby decreasing the life-threatening risk of the disease. Bromelain is the most effective protein-digesting enzyme when it comes to stimulating collagenase and breaking down collagen by dissolving the peptide bonds that hold their proteins together. The pineapple’s power should not be underestimated in effectively treating Peyronie’s as bromelain may even slow or reverse the tissue buildup that causes this disease [62].

### 6.14. Antiulcer Effect

Bromelain’s therapeutic efficacy has been proven against gastric ulcers in research with animals. In an extensive study of bromelain’s effect on the gastric mucosa, the uptake of radioactive sulfur was increased to 50%, and that of glucosamine was increased from 30% to 90%. In the case of gastric ulcers, the gastric mucosa can be healed more rapidly by increased uptake of these substances [12]. In a study, the use of bromelain mediated ulcer and Pylorus ligation model in albino rats was assessed for the Antiulcer activity. It demonstrates important dose-dependent ulcer-protective behavior. In EEAC and AEAC treated groups, in comparison with the ulcer control group, the ulcer index was reduced substantially. In ulcer control animals, the pH, free acidity, and total acidity levels were substantially reduced in the EEAC and AEAC treated groups, as compared to the ulcer control groups, and the pH of the Pylorus ligation model, the free acidity and total acidity levels were significantly decreased [111].

### 6.15. Postsurgery Recovery

The average number of recovery days following surgery can be reduced by administering bromelain to help reduce postsurgery inflammation [112,113]. Clinical trials illustrate that bromelain administration results in a reduction of bruising, pain, and swelling in the recovery time following episiotomy [88]. Sports injuries and acute inflammation can be treated successfully by bromelain therapy as well [17,96].

### 6.16. Antiasthmatic Effect

Asthma is characterized by inflammation of the airways, leading to amplifiedeosinophils and T lymphocyte levels in the bronchial mucosa and broncho-alveolar lavage (BAL) fluid [5,114]. Bromelain is used widely as a therapeutic tool for allergic airway disease (AAD) [5,115]. The total BAL leukocytes (eosinophils and lymphocytes) and cellular infiltrates are reduced by bromelain, thus relieving asthma symptoms [5,116]. Bromelain also prominently reduces BAL CD4^+^, CD8^+^, CD4^+^ T, and CD25^+^ T cells [5,117]; interleukins IL-4, IL-13, IL-12, IL-17, and IFN-α in the serum; and the ratio of CD4^+^ to CD8^+^ cells [5,115,116,118,119].

### 6.17. Treatment of Dermatological Disorders

#### 6.17.1. Pityriasis Lichenoides Chronica (PLC)

Pityriasis lichenoides chronica is a rare cutaneous disorder characterized by the development of multiple, scaly, erythematous to brown papules on the trunk and extremities [120]. During surgery, all PLC victims completely recovered with zero side effects, due to bromelain’s immunomodulatory and antiviral characteristics [121].

#### 6.17.2. Scleroderma

Bromelain has shown promise in the treatment of scleroderma, a condition marked by a gradual hardening of the skin and induration triggered by irregular connective tissue formation [122,123].

### 6.18. Immunogenicity

Bromelain and bromelain-sensitive molecules have been shown to eliminate T cell CD44 molecules from lymphocytes [53,124,125]. Munzig et al. (1994) found that CD44 expression was decreased about 10 times more by highly purified bromelain protease F9 than by crude bromelain, achieving approximately 97% inhibition of CD44 expression [13]. For the immunomodulatory role of protease therapy, proteolytic cleavage could serve as an additional aid. Following oral dosing, Hale (2002) found bromelain to exhibit strong immunogenicity [125]. Repeated exposure was required in further studies to produce antibromelain antibodies in a dose-dependent manner [126]. Bromelain enhanced T-cell-dependent, antigen-specific, B cell antibody responses at varied concentrations [63].

### 6.19. Antitumor Effect

Bromelain has shown tumor cell growth retardation in lung metastasis. Queensland Institute of Medical Research (QIMR) announced the discovery of two proteins—CCS and CCZ—that could inhibit the development of a wide variety of tumor cells, including those of melanoma and breast, lung, colon, and ovarian cancers. The research is underway, and these conclusions are currently not accurate [127]. In vitro studies have shown that platelets pretreated with bromelain in vitro lost their ability to induce several metastatic tumors’ invasiveness. In the meantime, it has been shown that metastasized cells bear CD44 adhesion molecules on their surface when migrating via the vessels, adhering to endothelial cells with the ligand hyaluron. It took twice as long for precancerous lesions to develop in the bromelain-treated sample instead of the control group. The QIMR researchers suggest that bromelain, in addition to its known proteolytic anticoagulant activities, may have other pharmacological applications. The research was conducted and published in the *Journal of Clinical Pharmacology and Biomarkers* (2005), at QIMR. However, as the research is still underway and they do not have access to all the study results, the author does not plan to make any current recommendations.

By proteolysis, bromelain cleaves CD44 molecules preferentially, thereby inhibiting the first steps of the metastatic process. Bromelain is capable of inhibiting both in situ and in vitro platelet aggregation and platelet-stimulated tumor cell invasiveness. However, to determine the structure and characteristics of the two compounds identified by QIMR researchers—CCS and CCZ—further study is required (QIMR, 2005).

**Table 1:** Therapeutic studies of bromelain based on experimental studies.

Fields of Study	Subjects	Dosage	Outcomes	References
Anti-inflammatory	Rats	10 and 20 mg/kg	Large reduction in exudate concentrations of both substance P and PGE2	[60]
Antimicrobial Activity	* Streptococcus mutans * , *Enterococcus fecalis*, Aggregatibacteractinomycetemcomitans (Aa), and Porphyromonasgingivalis	Minimum inhibitory concentration (MIC) of bromelain	* S. mutans * showed sensitivity at the lowest concentration of 2 mg/mL as compared to *E. fecalis* (31.25 mg/mL), while Pgingivalis showed sensitivity at the lowest concentration of 4.15 mg/mL as compared to Aa (16.6 mg/mL)	[128]
Antibiotic Potentiation	Rabbits	20–25mg/kg	Intramuscular and intraduodenal administration of bromelain enhanced penicillin-content of the cerebrospinal fluid, which normallyis much lower than in serum	[129,130]
Hepatic Microcirculation	140 Rats	0.1, 1.0, or 10 mg/kg	Increased leukocyte adherence, apoptosis rate, Kupffer cell activation, and endothelial cell damage, AST and ALT levels were significantly increased, improved microcirculation, increased eNOS expression	[131]
Anti-ulcer activity	Rats	200 ng/kg	Ulcer index and total acidity level were significantly reduced.	[111]
Anti-tumoral activity	Mice	12.5 and 25 mg/kg	Significantly decreased the amount of lung metastasis used by LLC transplantation	[46]
Anthelmintic efficacy	* Haemonchus contortus *	150 μM concentration	Important adulticidal action on *Haemonchus contortus* to destroy all worms, damage their cuticle after 8 h of incubation, and eventually cause worms to disintegrate	[105]
	Female CD1 mice	Different concentrations	Decreased amount of *Heligmosomoides polygyrus*	[132]
	Chickens	1008 mg/kg, 504 mg/kg, 255 mg/kg	Total worm count was significantly decreased	[133]
	Mice	0.2 mL containing 240 nmol stem bromelain	24.5% reduction in worm burdens	[134]
Anti-rheumatic activity	Rats	50, 100, 250 and 500 mg/kg	Significantly reduced the swelling in the paw of rats	[135]
Antinociceptive	48 Wistar rats	30 mg/kg and 50 mg/kg	The thermal hyperalgesia and allodynic mechanical indices of neuropathic pain were greatly reduced by bromelain	[75]
Immunomodulatory	Mice	200 mg/mL	Bromelain improved T-cell-dependent, Ag-specific, B cell antibody responses	[63]
Anti-platelet Activity	Rats	1, 5, 10, 20, and 30 mg/kg	Blood coagulation was delayed significantly	[17,130]

## 7. Bromelain and Nanoparticles: Application in Pharmaceutics

Nanoparticles (NPs), as used in pharmaceutics, have made a substantial contribution to the modern treatment of various ailments. Bromelain, as a proteolytic substance, promotes binding affinity and modifies the surface area of the particles. Currently, a few popular inorganic, synthetic, and natural compounds, as well as niosomes and lipid core nano-capsules, are widely used for biomedical purposes.

Mesoporous silica nanoparticles (MSNs) are biocompatible products with a wide range of surface area and pore volume and provide remarkable advantages for absorption and loading of drugs inside pores. Parodi and Haddix established MSNs with surface bromelain to increase the diffusion properties of NPs upon interaction with the ECM. Throughout the study, researchers also found that the modulation of bromelain improved particle affinity for the extracellular tumor matrix and demonstrated a minor influence on viable cells and the endolysosomal behavior of the cell. Bromelain–MSN showed that a Matrigel-like form was easily digested and diffused, and the endothelial cells were blocked by the ECM [11,136,137].

Gold is a well-documented metal that exerts biological actions to a greater extent than nanoparticles. Gold NPs perform diverse biomedical activities, including functionalization of the surface, which has helped develop new insights for anticancer and antimicrobial medications. However, as a unique disadvantage, the most common techniques for their production are harmful to the surroundings. The quest for bioactive compounds for the formation of gold NPs was thus promoted, and bromelain has proven to be both a reducing and capping agent. Bromelain-coated gold NPs were produced, as per the description of Khan, Danish Rizvi, as activators, bio-conjugated with levofloxacin using 1-ethyl-3-(3-dimethylamino-propyl)-carbodiimide [11,138,139].

Polyacrylic acid (PAA) is a water-soluble polymer that can be chemically changed by free groups of carboxyl, and several measures have been taken to build PAA-based particulate systems. Studies have been conducted to develop improved PAA with carbodiimide technology, and bromelain has been coupled with PAA NPs to conquer intestinal mucus gel. The method of conjugation with levofloxacin resulted in a product recovery of 59.2%, proving effective. However, as opposed to the indigenous bromelain production, enzyme conjugation on the polymeric scaffold induced a lack of enzyme activity, and the remaining enzymatic activity was estimated at 63.3% [11,140,141].

Chitosan is a mucopolysaccharide produced by the deacetylation of chitin, the main compound in crustacean exoskeletons. Chitosan nanoparticles can be produced using various methods. Studies have reported the uses of bromelain and its loading into nanoparticles derived from both natural and synthetic polymers [142]. Bromelain is not used exclusively as a coated active substance, but performs various roles in medicinal nanotechnology, including combining with chitosan. Bromelain acts as a surface modifier in Lactobionic acid-modified chitosan nanoparticles to improve their ability to penetrate tumor cells. Bromelain is also encapsulated using linoleic acid–modified carboxymethyl chitosan, enhancing its thermal properties and ensuring catalytic function [143].

## 8. Synergistic Effects of Bromelain

Pauzi et al. (2016) conducted a research regarding the synergistic effects of bromelain with cisplatin. In the study, MDA-MB-231 cells were treated with different bromelain or cisplatin concentrations (0.24–9.5 μM) and four different combining agents, to determine their individual and combined effects after 24 and 48 h. Using an MTT assay, cell viability was analyzed. An overview of the cell cycle and the Annexin-V-FITC assay was used for the induction of apoptosis. A JC-1 staining test was taken to assess the function of the mitochondrial membrane potential in the apoptotic process. Western blot analysis and proteome profiling using an antibody array kit was used to quantify apoptotic protein levels. The dose-and-time dependent decrease of MDA-MB-231 cells’ viability at 24 and 47 h resulted in single-agent cisplatin or bromelain therapy. Moreover, many of the combinations assessed in the present study exhibited synergistic effects on MDA-MB-231 cells after 48 h, which can be used to treat breast cancer [144].

The essential immunomodulatory functions of bromelain and curcumin interfere in the key phases of pathophysiology of the COVID-19. Their anti-inflammatory properties include transcription factors being inhibited and proinflammatory mediators subsequently decreased. Fibrinolytic and anticoagulant properties are also present. Bromelain also prevents cyclooxygenase, modulates prostaglandins and thromboxane, inflammation and coagulation, as well as bradykinin hydrolyses. Interestingly, curcumin has been shown in silico studies to prevent the entry of the severe acute respiratory syndrome coronavirus 2 (SARS-CoV-2) into cells, and recent testing has shown that bromelain can inhibit viral entry into cells as well. The absorption of curcumin following oral administration is significantly improved by bromelain [145]. Bromelain and Acetylcysteine (BromAc) take synergistic action against glycoproteins by splitting the glycosidic lines and disulfide linkages. SARS-CoV-2 proteins have been disrupted by BromAc. Acetylcysteine lowered the spike and envelope protein disulfide bonds [146].

Another study was investigated to understand the impacts of a combined anthocyanins and bromelain supplement (BE) on endothelial function, BP, TAC, oxygen utility capacity, and fatigability in healthy adults. Healthy adults obtained randomized crossover design of BE or placebo in the research. Pre- and post-BE and placebo consumption are tested for brachial flow-mediated dilation (FMD), BP, TAC, heart rating, oxygen capacity, and fatigability. In comparison to the placebo group, the BE group demonstrated a substantial improvement in FMD, decrease in systolic BP, and increased oxygen use capability. Saturation of tissue and oxidized Hb increased significantly during the intake of BE, while deoxidized Hb decreased significantly during the workout. Furthermore, after intake of BE, TAC was greatly improved [147].

## 9. Side Effects

### 9.1. Gastrointestinal Effects

The most frequent adverse effects confirmed in people who overdose on bromelain include vomiting, nausea, diarrhea, heart palpitations, digestive problems, lack of appetite, fatigue, body discomfort, dizziness, drowsiness, and lethargy. Uterine bleeding and heavy menstruation can occur as well. People having peptic ulcer and any other digestive problems should not intake bromelain in any form—they need to consult with health care professionals prior to using bromelain.

### 9.2. Allergic Reactions

Another significant side effect of bromelain is mild to severe allergic reaction affecting the skin or digestive system. Allergic reactions of the skin include hives, rash, itching, and swelling. Individuals may also suffer from breathing issues and tightness in the throat. People allergic to carrots, celery, fennel, rye, papaya, birch, or cypress pollen, certain grasses, or latex are more susceptible to these side effects.

### 9.3. Heavy Bleeding

Bromelain is evaluated to bleeding risk. Those who are suffering from and being treated for blood or bleeding disorders should use bromelain only after medical intervention and under strict health administration. Bromelain cannot be used for two or three weeks prior to any dental or surgical operation. It is not suitable for use during pregnancy and childbirth. Liver and kidney disorder sufferers should also avoid bromelain [13,148].

### 9.4. Possible Drug and Herb Interactions

Individuals taking antiplatelet or anticoagulant medications, including aspirin, heparin, warfarin (Coumadin), and clopidogrel (Plavix), as well as nonsteroidal anti-inflammatory drugs (NSAIDs) as well ibuprofen (Motrin, Advil) and naproxen (Naprosyn, Aleve) should use bromelain only under the supervision of a practitioner. Bromelain should be used with caution by people taking supplements and herbs that augment bleeding risk, for instance, garlic and *Ginkgo biloba*. Experiments have proposed that bromelain may possibly increase the absorption of medications—including antibiotics (such as tetracycline and amoxicillin); chemotherapy drugs (such as 5-fluorouracil and vincristine); blood pressure medications (specifically, ACE inhibitors, such as captopril (Capoten) and lisinopril (Zestril)); drugs (such as lorazepam (Ativan), benzodiazepines, or diazepam (Valium)) that induce drowsiness; certain antidepressants; opioids (including codeine); and barbiturates (such as phenobarbital) [15].

In some experimental trials, the average lethal dose of bromelain was higher than 10 g/kg when orally administered. The median lethal dosage was 20–35 mg/kg and 36.7–85.2 mg/kg, respectively, when given intravenously and intraperitoneally. There are few side effects recorded in most reports of bromelain. No severe adverse effects have been identified in a study of clinical trials examining the impact of bromelain on osteoarthritis, although there have been several cases of stomach symptoms, headache, exhaustion, dry mouth, skin rash, and unspecified allergic reactions. Bromelain has been delivered at dosages varying from 540 to 1890 mg/day in these trials. Compared with normal care, higher dosages of bromelain appeared to have higher incidences of harmful drug reactions. In other research, sporadic reports of allergic reactions and symptoms of asthma, related to occupational exposure to bromelain, have been identified. Adverse reactions resulted in most situations following bromelain intake; but, after a pineapple peroral contest, certain patients developed gastrointestinal distress. In these instances, respiratory and gastrointestinal effects were discovered to result from bromelain-mediated immunoglobulin E reactions. Some scholars also indicated that when used in conjunction with other drugs, such as aspirin and warfarin, the anticoagulant effects of bromelain may accelerate bleeding. Bromelain is commonly deemed healthy amid records of harmful effects, although it is necessary to remember that the majority of clinical trials have examined bromelain in adult samples. Therefore, no knowledge on the efficacy of bromelain is accessible for children under 18 years of age. Similarly, there is no evidence available on the efficacy of bromelain offered at higher concentrations, whether used in conjunction with other drugs or over lengthy periods of time. [149].

## 10. Toxicity

After daily administration of bromelain up to 750 mg/kg in dogs, no toxicity was reported after six months. The lethal dose LD_50_ is greater than 10 g/kg in mice [17,150]. No carcinogenic or teratogenic effects were observed when administered to rats with dosages of 1500 mg/kg per day. No change in food intake, or histology of heart, kidney, spleen, growth, or hematological parameters was provoked, due to bromelain administration [17,73]. In one study, Eckert et al. [42] found no significant changes in blood coagulation parameters after the intake of bromelain with doses up to 3000 FIP units per day.

## 11. Clinical Studies

Studies demonstrate that bromelain exhibits efficacious chemo-preventive properties and other studies include its side effects. These studies depict the therapeutic efficacy, as well as the minimal toxicity of bromelain. In a study, the anti-inflammatory property of bromelain was investigated. Bromelain lessened and reversed the pathologic effects of inflammation at a dose of 160 mg per day [151]. Bromelain is also used in the treatment of osteoarthritis. It reduced soft tissue swelling by 72.4% [152] and pain [72,153,154,155]. Postoperative pain, edema, and erythema were significantly lowered in another study group by using the prescribed amount of bromelain, which implies its significance in the treatment of chronic wounds [156]. Bromelain also induces allergic reactions by consuming 0.03 mg of bromelain per day [157], used in debridement of burns [91], treats trauma [158], and edema [159] at different doses. Among all the studies, the oral route is the most used and risk-free route of administration. The clinical studies of bromelain were summarized in Table 2.

## 12. Conclusions and Future Perspectives

Bromelain is considered to be a high-value enzyme in the therapeutics field as it is an effective treatment for inflammation, cancer, osteoarthritis, severe wounds, dental plaque, gingivitis, and various pathogens. As a natural and nontoxic compound, bromelain can be used as an alternative to multiple chemical ingredients and artificially manufactured medicines. Bromelain in pineapple is a type of enzyme known as a protease, which breaks other proteins apart by cutting the chains of amino acids. Bromelain selectively prevents proinflammatory prostaglandins’ biosynthesis obviously via indirect intervention. The sensitivity of the pineapple protease has been shown to be similar to the endogenous protease plasmin. The arachidonate cascade at the thromboxane synthetase level is assumed to be further inflamed with bromelains. Bromelain and curcumin interfere in the key phases of the pathophysiology of the COVID-19. Their anti-inflammatory properties include transcription factors being inhibited and proinflammatory mediators subsequently decreased. Bromelain also prevents cyclooxygenase, modulates prostaglandins and thromboxane, inflammation and coagulation, as well as bradykinin hydrolysis. Curcumin has been shown in silico studies to prevent the entry of the severe acute respiratory syndrome coronavirus 2 (SARS-CoV-2) into cells. Another study investigated the usefulness of combined anthocyanins and bromelains supplement (BE) on endothelial function, BP, TAC, oxygen utility capacity, and fatigability in healthy adults. It shows only mild side effects and has low toxicity; therefore, future researchers should develop more innovative extraction methods to expand the bromelain market. Bromelain should be segregated by adsorption and purified by nanoparticles. Bromelain exhibits multiaction capabilities in the field of pharmacology, and researchers should carry out further research to understand bromelain’s mechanism of action so health practitioners can take advantage of its benefits.

## Figures and Tables

**Table 2:** Clinical studies of bromelain based on experimental studies.

Fields of Study	Subjects	Dosage	Outcomes	References
Anti-inflammatory Activity	25 patients	160 mg/day	Reversed the pathologic effects of inflammation	[151]
Treatment of Osteoarthritis	29 moderate to severe arthritis patients	60–160 mg/day	Reduction in soft tissue swelling in 72.4%	[152]
	60 patients	540 mg/day	Sum score of various pain (active, pressure, rest, night) and dysfunction (four point category scale) measures	[72]
	73 patients	540 mg/day	Lequesne index (pain and function), Reduction in pain	[153]
	50 patients	1890 mg/day	Likert scale to assess pain and reduction in pain	[154]
	80 patients	945 mg/day	Mobility and pain reduction	[155]
Treatment of Chronic Wound	80 patients	Prescribed amount of bromelain	Postoperative pain, edema, and erythema were significantly lowered in the study group	[156]
Allergic Reactions	1 worker having contact with bromelain	0.03 mg/day	Skin and respiratory allergic reaction and nausea, dyspnea, distension, abdominal pain, and diarrhea	[157]
Debridement of Burns	154 patients	n.m.	Covered up to 67% TBSA, treated with DGD as a part of the burn care routine of this burn unit. The primary endpoints were percentage of eschar removed and time to wound closure	[91]
	20 hospitalized burn patients	1, 2, or 4 g in 20 mL of gel per 1% TBSA	Primarily, time to >95% wound closure or re-epithelialization. Finally, number of debridement procedures and percentage debridement of the burn eschar	[160]
	140 patients	Recommended dose	Covering up to 30%	[160]
Trauma	59 patients	n.m.	Reduced pain and swelling, early return to function	[158]
Dentistry	45 subjects	4 × 250 mg	Reduced erythema, pain, and inflammation	[108]
Anti-Edema	47 randomly selected patients	20-mg	The mean bleeding time decreased slightly (from 1.09 to 1.00 min) after a week of bromelain therapy	[159]

n.m.: not mentioned.

## Data Availability

Available data are presented in the manuscript.
