# Correction: Chakraborty et al. Bromelain a Potential Bioactive Compound: A Comprehensive Overview from a Pharmacological Perspective. *Life* 2021, *11*, 317

**DOI:** 10.3390/life14040483

**Published:** 2024-04-07

**Authors:** Arka Jyoti Chakraborty, Saikat Mitra, Trina E. Tallei, Abu Montakim Tareq, Firzan Nainu, Donatella Cicia, Kuldeep Dhama, Talha Bin Emran, Jesus Simal-Gandara, Raffaele Capasso

**Affiliations:** 1 Department of Pharmacy, Faculty of Pharmacy, University of Dhaka, Dhaka 1000, Bangladesh; arkwcky@gmail.com (A.J.C.); saikatmitradu@gmail.com (S.M.); 2 Department of Biology, Faculty of Mathematics and Natural Sciences, Universitas Sam Ratulangi, Manado 95115, North Sulawesi, Indonesia; trina_tallei@unsrat.ac.id; 3 Department of Pharmacy, International Islamic University Chittagong, Chittagong 4318, Bangladesh; montakim0.abu@gmail.com; 4 Faculty of Pharmacy, Hasanuddin University, Makassar 90245, Sulawesi Selatan, Indonesia; firzannainu@unhas.ac.id; 5 Department of Pharmacy, University of Naples Federico II, 80131 Naples, Italy; donatella.cicia@unina.it; 6 Division of Pathology, ICAR-Indian Veterinary Research Institute, Izatnagar, Bareilly 243122, Uttar Pradesh, India; 7 Department of Pharmacy, BGC Trust University Bangladesh, Chittagong 4381, Bangladesh; 8 Nutrition and Bromatology Group, Department of Analytical and Food Chemistry, Faculty of Food Science and Technology, University of Vigo—Ourense Campus, E32004 Ourense, Spain; 9 Department of Agricultural Sciences, University of Naples Federico II, 80055 Naples, Italy

## Text Correction

The authors were not aware of errors made in one small subsection (Section 6.17. Antidiarrheal Effect, including the data in the table of effects) of this paper [1], and, hence, wish to make the following corrections to this paper. 

The authors wish to delete Section 6.17: Antidiarrheal Effect, including the data in the table of effects, and reorganize the references. The deleted section appears as follows:

*6.17. Antidiarrheal Effect*

Diarrhea has long been a cause of death and illness in children and young animals [120,121,122]. Enterotoxigenic in ileus, *E. coli* (ETEC), and *Vibrio cholerae* are two primary microorganisms that cause diarrhea [123]. ETEC produces one or both of a heat-labile (LT) or heat-stable enterotoxin (either STa or STb), and *V. cholerae* releases cholera toxin (CT) [7]. Drugs, such as nicotinic acid, berberine sulfate, loperamide, and chlorpromazine, have been applied in animal models to inhibit the secretion of CT and LT [124,125,126]. Indomethacin, chlorpromazine, and berberine also decrease secretion induced by STa [124,126,127]. Despite the efficacy of these antisecretory compounds in animals, none are routinely available for use in humans because the doses required for efficacy are large enough to cause adverse side effects [128]. Over time, oral rehydration therapy was introduced, significantly improving patients’ mortality and morbidity with acute infectious diarrhea. Oral rehydration, however, does not hinder the secretion of toxins or alleviate diarrhea [129]. Scientists have demonstrated that bromelain has antidiarrheal properties [128,130]. A study by Mynott et al. (1997) used stem bromelain to demonstrate these antisecretory traits. The results showed that bromelain could prevent net changes in intestinal short-circuit current (Isc) using rabbit ileum installed in chambers, as well as mediate fluid secretion by secretagogues working via cAMP (cyclic-3′,5′-adenosine monophosphate), cGMP (cyclic-3′,5′-guanosine monophosphate), and calcium-dependent signaling pathways [131]. As one of these mechanisms is triggered by most toxins that induce diarrhea, bromelain is expected to be an important nutraceutical medication for this ailment. Bromelain was 62% effective in preventing LT-mediated secretion in this study, 51% effective against CT, and 35% effective against STa. Prostaglandin E2, theophylline, calcium-ionophore A23187, 8-Br-cAMP (8-bromo-cyclic-3′,5′-adenosine monophosphate), and 8-Br-cGMP (8-bromo-cyclic-3′,5′-guanosine monophosphate), well-known intracellular ion secretion mediators, also experienced secretory modifications. The effectiveness of bromelain has not been attributed to decreased tissue viability, due to its proteolytic effects on enterocytes, as shown by experiments measuring the absorption of nutrients into intestinal cells and others measuring the short-circuit response to glucose. A study performed by Roselli et al. (2007) on the impact of various plant extracts and natural substances (PENS) on ETEC-induced membrane damage in pig intestinal cells has shown that bromelain is among those with a protective effect [122].

## Error in Table

In the original publication, there was a mistake in Table 1. The data of reference [128] have been deleted. The corrected Table 1 appears below.

**Table 1:** Therapeutic studies of bromelain based on experimental studies.

Fields of Study	Subjects	Dosage	Outcomes	References
Anti-inflammatory	Rats	10 and 20 mg/kg	Large reduction in exudate concentrations of both substance P and PGE2	[60]
Antimicrobial Activity	*Streptococcus mutans*, *Enterococcus fecalis*, Aggregatibacteractinomycetemcomitans (Aa), and Porphyromonasgingivalis	Minimum inhibitory concentration (MIC) of bromelain	*S. mutans* showed sensitivity at the lowest concentration of 2 mg/mL as compared to *E. fecalis* (31.25 mg/mL), while Pgingivalis showed sensitivity at the lowest concentration of 4.15 mg/mL as compared to Aa (16.6 mg/mL)	[128]
Antibiotic Potentiation	Rabbits	20–25mg/kg	Intramuscular and intraduodenal administration of bromelain enhanced penicillin-content of the cerebrospinal fluid, which normallyis much lower than in serum	[129,130]
Hepatic Microcirculation	140 Rats	0.1, 1.0, or 10 mg/kg	Increased leukocyte adherence, apoptosis rate, Kupffer cell activation, and endothelial cell damage, AST and ALT levels were significantly increased, improved microcirculation, increased eNOS expression	[131]
Anti-ulcer activity	Rats	200 ng/kg	Ulcer index and total acidity level were significantly reduced.	[111]
Anti-tumoral activity	Mice	12.5 and 25 mg/kg	Significantly decreased the amount of lung metastasis used by LLC transplantation	[46]
Anthelmintic efficacy	*Haemonchus contortus*	150 μM concentration	Important adulticidal action on *Haemonchus contortus* to destroy all worms, damage their cuticle after 8 h of incubation, and eventually cause worms to disintegrate	[105]
	Female CD1 mice	Different concentrations	Decreased amount of *Heligmosomoides polygyrus*	[132]
	Chickens	1008 mg/kg, 504 mg/kg, 255 mg/kg	Total worm count was significantly decreased	[133]
	Mice	0.2 mL containing 240 nmol stem bromelain	24.5% reduction in worm burdens	[134]
Anti-rheumatic activity	Rats	50, 100, 250 and 500 mg/kg	Significantly reduced the swelling in the paw of rats	[135]
Antinociceptive	48 Wistar rats	30 mg/kg and 50 mg/kg	The thermal hyperalgesia and allodynic mechanical indices of neuropathic pain were greatly reduced by bromelain	[75]
Immunomodulatory	Mice	200 mg/mL	Bromelain improved T-cell-dependent, Ag-specific, B cell antibody responses	[63]
Anti-platelet Activity	Rats	1, 5, 10, 20, and 30 mg/kg	Blood coagulation was delayed significantly	[17,130]

## Revised References

1. Heinicke, R.M.; Gortner, W.A. Stem bromelain—A new protease preparation from pineapple plants. *Econ. Bot.* **1957**, *11*, 225–234. https://doi.org/10.1007/BF02860437.
2. Hatano, K.I.; Kojima, M.; Tanokura, M.; Takahashi, K. Solution structure of bromelain inhibitor VI from pineapple stem: Structural similarity with Bowman—Birk trypsin/chymotrypsin inhibitor from soybean. *Biochemistry* **1996**, *35*, 5379–5384. https://doi.org/10.1021/bi952754.
3. Tayab, M.A.; Chowdhury, K.A.A.; Jabed, M.; Mohammed Tareq, S.; Kamal, A.T.M.M.; Islam, M.N.; Uddin, A.M.K.; Hossain, M.A.; Emran, T.B.; Simal-Gandara, J. Antioxidant-Rich Woodfordia fruticosa Leaf Extract Alleviates Depressive-Like Behaviors and Impede Hyperglycemia. *Plants* **2021**, *10*, 287. https://doi.org/10.3390/plants10020287.
4. Mamo, J.; Assefa, F. Antibacterial and Anticancer Property of Bromelain: A Plant Protease Enzyme from Pineapples (*Ananas comosus*). *Curr. Trends Biomed. Eng. Biosci.* **2019**, *19*, 60–68.
5. Manzoor, Z.; Nawaz, A.; Mukhtar, H.; Haq, I. Bromelain: Methods of Extraction, Purification and Therapeutic Applications. *Braz. Arch. Biol. Technol.* **2016**, *59*, e16150010. https://doi.org/10.1590/1678-4324-2016150010.
6. Bhui, K.; Prasad, S.; George, J.; Shukla, Y. Bromelain inhibits COX-2 expression by blocking the activation of MAPK regulated NF-kappa B against skin tumor-initiation triggering mitochondrial death pathway. *Cancer Lett.* **2009**, *282*, 167–176. https://doi.org/10.1016/j.canlet.2009.03.003.
7. Mynott, T.L.; Ladhams, A.; Scarmato, P.; Engwerda, C.R. Bromelain, from pineapple stems, proteolytically blocks activation of extracellular regulated kinase-2 in T cells. *J. Immunol.* **1999**, *163*, 2568–2575.
8. Bhattacharyya, B.K. Bromelain: An overview. *Indian J. Nat. Prod. Resour.* **2008**, *7*, 359–363.
9. Chobotova, K.; Vernallis, A.B.; Majid, F.A.A. Bromelain’s activity and potential as an anti-cancer agent: Current evidence and perspectives. *Cancer Lett.* **2010**, *290*, 148–156. https://doi.org/10.1016/j.canlet.2009.08.001.
10. Tochi, B.N.; Wang, Z.; Xu, S.Y.; Zhang, W. Therapeutic application of pineapple protease (Bromelain): A review. *Pak. J. Nutr.* **2008**, *7*, 513–520. https://doi.org/10.3923/pjn.2008.513.520.
11. Ataide, J.A.; Gérios, E.F.; Mazzola, P.G.; Souto, E.B. Bromelain-loaded nanoparticles: A comprehensive review of the state of the art. *Adv. Colloid Interface Sci.* **2018**, *254*, 48–55. https://doi.org/10.1016/j.cis.2018.03.006.
12. Dighe, N.S.; Pattan, S.R.; Merekar, A.N.; Laware, R.B.; Bhawar, S.B.; Nirmal, S.N.; Gaware, V.M.; Hole, M.B.; Musmade, D.S. Bromelain A Wonder Supplement: A Review. *Pharmacologyonline* **2010**, *1*, 11–18.
13. Munzig, E.; Eckert, K.; Harrach, T.; Graf, H.; Maurer, H.R. Bromelain protease F9 reduces the CD44 mediated adhesion of human peripheral blood lymphocytes to human umbilical vein endothelial cells. *FEBS Lett.* **1994**, *351*, 215–218. https://doi.org/10.1016/0014-5793(94)00860-4.
14. Jyoti, M.A.; Barua, N.; Hossain, M.S.; Hoque, M.; Bristy, T.A.; Mahmud, S.; Kamruzzaman; Adnan, M.; Chy, M.N.U.; Paul, A.; et al. Unravelling the biological activities of the Byttneria pilosa leaves using experimental and computational approaches. *Molecules* **2020**, *25*, 4737. https://doi.org/10.3390/molecules25204737.
15. Houck, J.C.; Chang, C.M.; Klein, G. Isolation of an effective debriding agent from the stems of pineapple plants. *Int. J. Tissue React.* **1983**, *5*, 125–134.
16. Larocca, M.; Rossano, R.; Santamaria, M.; Riccio, P. Analysis of pineapple [*Ananas comosus* (L.) Merr.] fruit proteinases by 2-D zymography and direct identification of the major zymographic spots by mass spectrometry. *Food Chem.* **2010**, *123*, 1334–1342. https://doi.org/10.1016/j.foodchem.2010.06.016.
17. Pavan, R.; Jain, S.; Shraddha; Kumar, A. Properties and Therapeutic Application of Bromelain: A Review. *Biotechnol. Res. Int.* **2012**, *2012*, 976203. https://doi.org/10.1155/2012/976203.
18. Corzo, C.A.; Waliszewski, K.N.; Welti-Chanes, J. Pineapple fruit bromelain affinity to different protein substrates. *Food Chem.* **2012**, *133*, 631–635. https://doi.org/10.1016/j.foodchem.2011.05.119.
19. Harrach, T.; Eckert, K.; Maurer, H.R.; Machleidt, I.; Machleidt, W.; Nuck, R. Isolation and characterization of two forms of an acidic bromelain stem proteinase. *J. Protein Chem.* **1998**, *17*, 351–361. https://doi.org/10.1023/A:1022507316434.
20. Illanes, A. Enzyme Production. In *Enzyme Biocatalysis: Principles and Applications*; Illanes, A., Ed.; Springer: Dordrecht, The Netherlands, 2008; pp. 57–106, ISBN 978-1-4020-8361-7.
21. Abreu, D.C.A.; De Figueiredo, K.C.S. Bromelain separation and purification processes from pineapple extract. *Braz. J. Chem. Eng.* **2019**, *36*, 1029–1039. https://doi.org/10.1590/0104-6632.20190362s20180417.
22. Benucci, I.; Liburdi, K.; Garzillo, A.M.V.; Esti, M. Bromelain from pineapple stem in alcoholic-acidic buffers for wine application. *Food Chem.* **2011**, *124*, 1349–1353. https://doi.org/10.1016/j.foodchem.2010.07.087.
23. Manderson, D.; Dempster, R.; Chisti, Y. Production of an active recombinant Aspin antigen in Escherichia coli for identifying animals resistant to nematode infection. *Enzyme Microb. Technol.* **2006**, *38*, 591–598. https://doi.org/10.1016/j.enzmictec.2005.03.029.
24. Muntari, B.; Amid, A.; Mel, M.; Jami, M.S.; Salleh, H.M. Recombinant bromelain production in *Escherichia coli*: Process optimization in shake flask culture by response surface methodology. *AMB Express* **2012**, *2*, 12. https://doi.org/10.1186/2191-0855-2-12.
25. Dutta, T.; Paul, A.; Majumder, M.; Sultan, R.A.; Emran, T.B. Pharmacological evidence for the use of Cissus assamica as a medicinal plant in the management of pain and pyrexia. *Biochem. Biophys. Rep.* **2020**, *21*, 100715. https://doi.org/10.1016/j.bbrep.2019.100715.
26. Maurer, H.R. Bromelain: Biochemistry pharmacology and medical use. *Cell. Mol. Life Sci.* **2001**, *58*, 1234–1245. https://doi.org/10.1007/PL00000936.
27. Harrach, T.; Eckert, K.; Schulze-Forster, K.; Nuck, R.; Grunow, D.; Maurer, H.R. Isolation and partial characterization of basic proteinases from stem bromelain. *J. Protein Chem.* **1995**, *14*, 41–52. https://doi.org/10.1007/BF01902843.
28. Hidayat, M.; Prahastuti, S.; Wargasetia, T.; Nugraha, K.; Soemardji, A.; Rahmawati, S.; Suliska, N.; Hasan, K. Green peas protein hydrolyzed by bromelain in simple procedure to improve kidney function in cisplatin-induced rats. *J. Rep. Pharm. Sci.* **2019**, *8*, 68–77. https://doi.org/10.4103/jrptps.jrptps_15_17.
29. Steinkraus, K.H. The use of bromelain in the hydrolysis of mackerel and the investigation of fermented fish aroma. *Int. J. Food Sci. Technol.* **1976**, *11*, 379–388. https://doi.org/10.1111/j.1365-2621.1976.tb00736.x.
30. Babu, B.R.; Rastogi, N.K.; Raghavarao, K.S.M.S. Liquid-liquid extraction of bromelain and polyphenol oxidase using aqueous two-phase system. *Chem. Eng. Process. Process. Intensif.* **2008**, *47*, 83–89. https://doi.org/10.1016/j.cep.2007.08.006.
31. Ketnawa, S.; Chaiwut, P.; Rawdkuen, S. Aqueous two-phase extraction of bromelain from pineapple peels (‘Phu Lae’ cultv.) and its biochemical properties. *Food Sci. Biotechnol.* **2011**, *20*, 1219–1226. https://doi.org/10.1007/s10068-011-0168-5.
32. Krishna, S.H.; Srinivas, N.D.; Raghavarao, K.S.; Karanth, N.G. Reverse micellar extraction for downstream processing of proteins/enzymes. *Adv. Biochem. Eng. Biotechnol.* **2002**, *75*, 119–183. https://doi.org/10.1007/3-540-44604-4_5.
33. Ahmed, S.; Rakib, A.; Islam, M.A.; Khanam, B.H.; Faiz, F.B.; Paul, A.; Chy, M.N.U.; Bhuiya, N.M.A.; Uddin, M.M.N.; Ullah, S.A. In vivo and in vitro pharmacological activities of Tacca integrifolia rhizome and investigation of possible lead compounds against breast cancer through in silico approaches. *Clin. Phytosci.* **2019**, *5*, 36. https://doi.org/10.1186/s40816-019-0127-x.
34. Mezzanotte, V.; Castiglioni, F.; Todeschini, R.; Pavan, M. Study on anaerobic and aerobic degradation of different non-ionic surfactants. *Bioresour. Technol.* **2003**, *87*, 87–91. https://doi.org/10.1016/S0960-8524(02)00211-0.
35. Umesh Hebbar, H.; Sumana, B.; Raghavarao, K.S.M.S. Use of reverse micellar systems for the extraction and purification of bromelain from pineapple wastes. *Bioresour. Technol.* **2008**, *99*, 4896–4902. https://doi.org/10.1016/j.biortech.2007.09.038.
36. Hebbar, U.H.; Sumana, B.; Hemavathi, A.B.; Raghavarao, K.S.M.S. Separation and Purification of Bromelain by Reverse Micellar Extraction Coupled Ultrafiltration and Comparative Studies with Other Methods. *Food Bioprocess Technol.* **2012**, *5*, 1010–1018. https://doi.org/10.1007/s11947-010-0395-4.
37. Hung, T.H.; Chang, Y.M.; Sung, H.Y.; Chang, C.T. Purification and characterization of hydrolase with chitinase and chitosanase activity from commercial stem bromelain. *J. Agric. Food Chem.* **2002**, *50*, 4666–4673. https://doi.org/10.1021/jf0114886.
38. Arumugam, A.; Ponnusami, V. Pineapple fruit bromelain recovery using recyclable functionalized ordered mesoporous silica synthesized from sugarcane leaf ash. *Braz. J. Chem. Eng.* **2013**, *30*, 477–486. https://doi.org/10.1590/S0104-66322013000300006.
39. Swaroop, G.; Viswanathan, G. Isolation and Characterization of Bromelain (BML) Proteases from Ananas cosmosus an asset to Cancer Chemotherapy. *Int. J. Pharmacol. Toxicol.* **2013**, *1*, 82–90. https://doi.org/10.14419/ijpt.v1i2.1397.
40. Biswas, F.B.; Roy, T.G.; Rahman, M.A.; Emran, T.B. An in vitro antibacterial and antifungal effects of cadmium(II) complexes of hexamethyltetraazacyclotetradecadiene and isomers of its saturated analogue. *Asian Pac. J. Trop. Med.* **2014**, *7*, S534–S539. https://doi.org/10.1016/S1995-7645(14)60286-8.
41. Costa, H.B.; Delboni, S.G.; Fortunato, F.S.; Venturaa, J.A. Proteolytic Activity in Stems of ‘Vitória’, ‘Smooth Cayenne’ and ‘Pérola’ Pineapple Plants. *Acta Hortic.* **2009**, *822*, 239–243. https://doi.org/10.17660/actahortic.2009.822.29.
42. Devakate, R.V.; Patil, V.V.; Waje, S.S.; Thorat, B.N. Purification and drying of bromelain. *Sep. Purif. Technol.* **2009**, *64*, 259–264. https://doi.org/10.1016/j.seppur.2008.09.012.
43. Yin, L.; Sun, C.K.; Han, X.; Xu, L.; Xu, Y.; Qi, Y.; Peng, J. Preparative purification of bromelain (EC 3.4.22.33) from pineapple fruit by high-speed counter-current chromatography using a reverse-micelle solvent system. *Food Chem.* **2011**, *129*, 925–932. https://doi.org/10.1016/j.foodchem.2011.05.048.
44. Taussig, S.J. The mechanism of the physiological action of bromelain. *Med. Hypotheses* **1980**, *6*, 99–104. https://doi.org/10.1016/0306-9877(80)90038-9.
45. Rathnavelu, V.; Alitheen, N.B.; Sohila, S.; Kanagesan, S.; Ramesh, R. Potential role of bromelain in clinical and therapeutic applications (Review). *Biomed. Rep.* **2016**, *5*, 283–288. https://doi.org/10.3892/br.2016.720.
46. Béez, R.; Lopes, M.T.P.; Salas, C.E.; Hernández, M. In vivo antitumoral activity of stem pineapple (*Ananas comosus*) bromelain. *Planta Med.* **2007**, *73*, 1377–1383. https://doi.org/10.1055/s-2007-990221.
47. Al Mahmud, Z.; Qais, N.; Bachar, S.C.; Hasan, C.M.; Emran, T.B.; Uddin, M.M.N. Phytochemical investigations and antioxidant potential of leaf of *Leea macrophylla* (Roxb.). *BMC Res. Notes* **2017**, *10*, 245. https://doi.org/10.1186/s13104-017-2503-2.
48. Juhasz, B.; Thirunavukkarasu, M.; Pant, R.; Zhan, L.; Penumathsa, S.V.; Secor, E.R.; Srivastava, S.; Raychaudhuri, U.; Menon, V.P.; Otani, H.; et al. Bromelain induces cardioprotection against ischemia-reperfusion injury through Akt/FOXO pathway in rat myocardium. *Am. J. Physiol. Heart Circ. Physiol.* **2008**, *294*, H1365–H1370. https://doi.org/10.1152/ajpheart.01005.2007.
49. Vilanova Neta, J.L.; Da Silva Lédo, A.; Lima, A.A.B.; Santana, J.C.C.; Leite, N.S.; Ruzene, D.S.; Silva, D.P.; De Souza, R.R. Bromelain enzyme from pineapple: In vitro activity study under different micropropagation conditions. *Appl. Biochem. Biotechnol.* **2012**, *168*, 234–246. https://doi.org/10.1007/s12010-012-9753-1.
50. Bhui, K.; Tyagi, S.; Prakash, B.; Shukla, Y. Pineapple bromelain induces autophagy, facilitating apoptotic response in mammary carcinoma cells. *BioFactors* **2010**, *36*, 474–482. https://doi.org/10.1002/biof.121.
51. Dhandayuthapani, S.; Perez, H.D.; Paroulek, A.; Chinnakkannu, P.; Kandalam, U.; Jaffe, M.; Rathinavelu, A. Bromelain-induced apoptosis in GI-101A breast cancer cells. *J. Med. Food* **2012**, *15*, 344–349. https://doi.org/10.1089/jmf.2011.0145.
52. Bhui, K.; Tyagi, S.; Srivastava, A.K.; Singh, M.; Roy, P.; Singh, R.; Shukla, Y. Bromelain inhibits nuclear factor kappa-B translocation, driving human epidermoid carcinoma A431 and melanoma A375 cells through G 2/M arrest to apoptosis. *Mol. Carcinog.* **2012**, *51*, 231–243. https://doi.org/10.1002/mc.20769.
53. Harrach, T.; Gebauer, F.; Eckert, K.; Kunze, R.; Maurer, H. Bromelain proteinases modulate the cd44 expression on human molt-4/8 leukemia and sk-mel-28 melanoma-cells in-vitro. *Int. J. Oncol.* **1994**, *5*, 485–488.
54. Paschke, S.; Jafarov, S.; Staib, L.; Kreuser, E.D.; Maulbecker-Armstrong, C.; Roitman, M.; Holm, T.; Harris, C.C.; Link, K.H.; Kornmann, M. Are colon and rectal cancer two different tumor entities? A proposal to abandon the term colorectal cancer. *Int. J. Mol. Sci.* **2018**, *19*, 2577. https://doi.org/10.3390/ijms19092577.
55. Chang, T.C.; Wei, P.L.; Makondi, P.T.; Chen, W.T.; Huang, C.Y.; Chang, Y.J. Bromelain inhibits the ability of colorectal cancer cells to proliferate via activation of ROS production and autophagy. *PLoS ONE* **2019**, *14*, e210274. https://doi.org/10.1371/journal.pone.0210274.
56. Higashi, T.; Kogo, T.; Sato, N.; Hirotsu, T.; Misumi, S.; Nakamura, H.; Iohara, D.; Onodera, R.; Motoyama, K.; Arima, H. Efficient Anticancer Drug Delivery for Pancreatic Cancer Treatment Utilizing Supramolecular Polyethylene-Glycosylated Bromelain. *ACS Appl. Bio Mater.* **2020**, *3*, 3005–3014. https://doi.org/10.1021/acsabm.0c00070.
57. Pillai, K.; Mekkawy, A.H.; Akhter, J.; Badar, S.; Dong, L.; Liu, A.I.; Morris, D.L. Enhancing the potency of chemotherapeutic agents by combination with bromelain and N-acetylcysteine—An in vitro study with pancreatic and hepatic cancer cells. *Am. J. Transl. Res.* **2020**, *12*, 7404–7419.
58. Rahaman, M.M.; Rakib, A.; Mitra, S.; Tareq, A.M.; Emran, T.B.; Shahid-Ud-daula, A.F.M.; Amin, M.N.; Simal-Gandara, J. The genus curcuma and inflammation: Overview of the pharmacological perspectives. *Plants* **2021**, *10*, 63. https://doi.org/10.3390/plants10010063.
59. Huang, J.R.; Wu, C.C.; Hou, R.C.W.; Jeng, K.C. Bromelain inhibits lipopolysaccharide-induced cytokine production in human THP-1 monocytes via the removal of CD14. *Immunol. Investig*. **2008**, *37*, 263–277. https://doi.org/10.1080/08820130802083622.
60. Gaspani, L.; Limiroli, E.; Ferrario, P.; Bianchi, M. In vivo and in vitro effects of bromelain on PGE2 and SP concentrations in the inflammatory exudate in rats. *Pharmacology* **2002**, *65*, 83–86. https://doi.org/10.1159/000056191.
61. Emran, T.B.; Rahman, M.A.; Uddin, M.M.N.; Rahman, M.M.; Uddin, M.Z.; Dash, R.; Layzu, C. Effects of organic extracts and their different fractions of five Bangladeshi plants on in vitro thrombolysis. *BMC Complement. Altern. Med.* **2015**, *15*, 128. https://doi.org/10.1186/s12906-015-0643-2.
62. Engwerda, C.R.; Andrew, D.; Murphy, M.; Mynott, T.L. Bromelain activates murine macrophages and natural killer cells in vitro. *Cell. Immunol.* **2001**, *210*, 5–10. https://doi.org/10.1006/cimm.2001.1793.
63. Engwerda, C.R.; Andrew, D.; Ladhams, A.; Mynott, T.L. Bromelain modulates T cell and B cell immune responses in vitro and in vivo. *Cell. Immunol.* **2001**, *210*, 66–75. https://doi.org/10.1006/cimm.2001.1807.
64. Barth, H.; Guseo, A.; Klein, R. In vitro study on the immunological effect of bromelain and trypsin on mononuclear cells from humans. *Eur. J. Med. Res.* **2005**, *10*, 325–331.
65. Kane, S.; Goldberg, M.J. Use of bromelain for mild ulcerative colitis. *Ann. Intern. Med.* **2000**, *132*, 680. https://doi.org/10.7326/0003-4819-132-8-200004180-00026.
66. Hale, L.P.; Greer, P.K.; Trinh, C.T.; Gottfried, M.R. Treatment with oral bromelain decreases colonic inflammation in the IL-10-deficient murine model of inflammatory bowel disease. *Clin. Immunol.* **2005**, *116*, 135–142. https://doi.org/10.1016/j.clim.2005.04.011.
67. Onken, J.E.; Greer, P.K.; Calingaert, B.; Hale, L.P. Bromelain treatment decreases secretion of pro-inflammatory cytokines and chemokines by colon biopsies in vitro. *Clin. Immunol.* **2008**, *126*, 345–352. https://doi.org/10.1016/j.clim.2007.11.002.
68. Stopper, H.; Schinzel, R.; Sebekova, K.; Heidland, A. Genotoxicity of advanced glycation end products in mammalian cells. *Cancer Lett.* **2003**, *190*, 151–156. https://doi.org/10.1016/S0304-3835(02)00626-2.
69. Rahman, M.A.; bin Imran, T.; Islam, S. Antioxidative, antimicrobial and cytotoxic effects of the phenolics of Leea indica leaf extract. *Saudi J. Biol. Sci.* **2013**, *20*, 213–225. https://doi.org/10.1016/j.sjbs.2012.11.007.
70. Subramaniam, V.; Gardner, H.; Jothy, S. Soluble CD44 secretion contributes to the acquisition of aggressive tumor phenotype in human colon cancer cells. *Exp. Mol. Pathol.* **2007**, *83*, 341–346. https://doi.org/10.1016/j.yexmp.2007.08.007.
71. Bierie, B.; Moses, H.L. Tumour microenvironment—TGFΒ: The molecular Jekyll and Hyde of cancer. *Nat. Rev. Cancer* **2006**, *6*, 506–520. https://doi.org/10.1038/nrc1926.
72. Leipner, J.; Iten, F.; Saller, R. Therapy with proteolytic enzymes in rheumatic disorders. *BioDrugs* **2001**, *15*, 779–789. https://doi.org/10.2165/00063030-200115120-00001.
73. Moss, J.N.; Frazier, C.V.; Martin, G.J. Bromelains. the Pharmacology of the Enzymes. *Arch. Int. Pharmacodyn. Thérapie* **1963**, *145*, 166–189.
74. Giacca, S. Clinical Experiences on the Action of Bromelin in Peripheral Venous Diseases and in Chronic Bronchitic States. *Minerva Med.* **1964**, *55*, 3925–3928.
75. Bakare, A.O.; Owoyele, B.V. Antinociceptive and neuroprotective effects of bromelain in chronic constriction injury-induced neuropathic pain in Wistar rats. *Korean J. Pain* **2020**, *33*, 13–22. https://doi.org/10.3344/kjp.2020.33.1.13.
76. De Giuli, M.; Pirotta, F. Bromelain interaction with some protease inhibitors and rabbit specific antiserum. *Drugs Exp. Clin.* **1978**, *4*, 21–23.
77. Jahan, I.; Tona, M.R.; Sharmin, S.; Sayeed, M.A.; Tania, F.Z.; Paul, A.; Chy, M.; Uddin, N.; Rakib, A.; Emran, T.B. GC-MS phytochemical profiling, pharmacological properties, and in silico studies of Chukrasia velutina leaves: A novel source for bioactive agents. *Molecules* **2020**, *25*, 3536. https://doi.org/10.3390/molecules25153536.
78. Brakebusch, M.; Wintergerst, U.; Petropoulou, T.; Notheis, G.; Husfeld, L.; Belohradsky, B.H.; Adam, D. Bromelain is an accelerator of phagocytosis, respiratory burst and Killing of Candida albicans by human granulocytes and monocytes. *Eur. J. Med. Res.* **2001**, *6*, 193–200.
79. Sartini, S.; Permana, A.D.; Mitra, S.; Tareq, A.M.; Salim, E.; Ahmad, I.; Harapan, H.; Emran, T.B.; Nainu, F. Current State and Promising Opportunities on Pharmaceutical Approaches in the Treatment of Polymicrobial Diseases. *Pathogens* **2021**, *10*, 245. https://doi.org/10.3390/pathogens10020245.
80. Massimiliano, R.; Pietro, R.; Paolo, S.; Sara, P.; Michele, F. Role of bromelain in the treatment of patients with pityriasis lichenoides chronica. *J. Dermatolog. Treat.* **2007**, *18*, 219–222. https://doi.org/10.1080/09546630701299147.
81. Uddin, M.Z.; Paul, A.; Rakib, A.; Sami, S.A.; Mahmud, S.; Rana, M.S.; Hossain, S.; Tareq, A.M.; Dutta, M.; Emran, T.B.; et al. Chemical Profiles and Pharmacological Properties with In Silico Studies on Elatostema papillosum Wedd. *Molecules* **2021**, *26*, 809. https://doi.org/10.3390/molecules26040809.
82. Taussig, S.J.; Batkin, S. Bromelain, the enzyme complex of pineapple (*Ananas comosus*) and its clinical application. An update. *J. Ethnopharmacol.* **1988**, *22*, 191–203. https://doi.org/10.1016/0378-8741(88)90127-4.
83. Rakib, A.; Ahmed, S.; Islam, M.A.; Uddin, M.M.N.; Paul, A.; Chy, M.N.U.; Emran, T.B.; Seidel, V. Pharmacological studies on the antinociceptive, anxiolytic and antidepressant activity of Tinospora crispa. *Phytother. Res.* **2020**, *34*, 2978–2984. https://doi.org/10.1002/ptr.6725.
84. Errasti, M.E.; Prospitti, A.; Viana, C.A.; Gonzalez, M.M.; Ramos, M.V.; Rotelli, A.E.; Caffini, N.O. Effects on fibrinogen, fibrin, and blood coagulation of proteolytic extracts from fruits of Pseudananas macrodontes, Bromelia balansae, and B. hieronymi (Bromeliaceae) in comparison with bromelain. *Blood Coagul. Fibrinolysis* **2016**, *27*, 441–449. https://doi.org/10.1097/MBC.0000000000000531.
85. Kelly, G.S. Bromelain: A literature review and discussion of its therapeutic applications. *Altern. Med. Rev.* **1996**, *1*, 243–257.
86. Ratnaningsih, D.A.; Subiyandono; Sri, W. The Effectiveness of Waste Crude Bromelain Pineapple and Papaya Fruit Mixture as Anti-Plaque Toothpaste. *J. Med. Sci. Clin. Res.* **2018**, *6*, 1–7. https://doi.org/10.18535/jmscr/v6i2.01.
87. Harmely, F.; Lucida, H.; Mukhtar, M.H. Efektifitas Bromelain Kasar dari Batang Nenas (*Ananas comosus* L. Merr) sebagai Antiplak dalam Pasta Gigi. *Sci. J. Farm. Dan Kesehat.* **2015**, *1*, 14. https://doi.org/10.36434/scientia.v1i1.11.
88. Howat, R.C.L.; Lewis, G.D. The Effect of Bromelain Therapy on Episiotomy Wounds—A Double Blind Controlled Clinical Trial. *BJOG Int. J. Obstet. Gynaecol.* **1972**, *79*, 951–953. https://doi.org/10.1111/j.1471-0528.1972.tb12194.x.
89. Singer, A.J.; McClain, S.A.; Taira, B.R.; Rooney, J.; Steinhauff, N.; Rosenberg, L. Rapid and selective enzymatic debridement of porcine comb burns with bromelain-derived Debrase^®^: Acute-phase preservation of noninjured tissue and zone of stasis. *J. Burn Care Res.* **2010**, *31*, 304–309. https://doi.org/10.1097/BCR.0b013e3181d0f4d4.
90. Krieger, Y.; Rosenberg, L.; Lapid, O.; Glesinger, R.; Bogdanov-Berezovsky, A.; Silberstein, E.; Sagi, A.; Judkins, K. Escharotomy using an enzymatic debridement agent for treating experimental burn-induced compartment syndrome in an animal model. *J. Trauma Inj. Infect. Crit. Care* **2005**, *58*, 1259–1264. https://doi.org/10.1097/01.TA.0000169867.08607.F1.
91. Rosenberg, L.; Lapid, O.; Bogdanov-Berezovsky, A.; Glesinger, R.; Krieger, Y.; Silberstein, E.; Sagi, A.; Judkins, K.; Singer, A.J. Safety and efficacy of a proteolytic enzyme for enzymatic burn debridement: A preliminary report. *Burns* **2004**, *30*, 843–850.
92. Banu, N.; Alam, N.; Islam, M.N.; Islam, S.; Sakib, S.A.; Hanif, N.B.; Chowdhury, M.R.; Tareq, A.M.; Chowdhury, K.H.; Jahan, S.; et al. Insightful Valorization of the Biological Activities of Pani Heloch Leaves through Experimental and Computer-Aided Mechanisms. *Molecules* **2020**, *25*, 5153. https://doi.org/10.3390/molecules25215153.
93. Lawrence, R.C.; Helmick, C.G.; Arnett, F.C.; Deyo, R.A.; Felson, D.T.; Giannini, E.H.; Heyse, S.P.; Hirsch, R.; Hochberg, M.C.; Hunder, G.G.; et al. Estimates of the prevalence of arthritis and selected musculoskeletal disorders in the United States. *Arthritis Rheum.* **1998**, *41*, 778–799. https://doi.org/10.1002/1529-0131(199805)41:5<778::AID-ART4>3.0.CO;2-V.
94. Bodi, T. The Effects of Oral Bromelains on Tissue Permeability to Antibiotics and Pain Response to Bradykinin: Double Blind Studies on Human Subjects. *Clin. Med.* **1966**, *73*, 61–65.
95. Kumakura, S.; Yamashita, M.; Tsurufuji, S. Effect of bromelain on kaolin-induced inflammation in rats. *Eur. J. Pharmacol.* **1988**, *150*, 295–301. https://doi.org/10.1016/0014-2999(88)90010-6.
96. Brien, S.; Lewith, G.; Walker, A.; Hicks, S.M.; Middleton, D. Bromelain as a Treatment for Osteoarthritis: A Review of Clinical Studies. *Evid. Based Complement. Altern. Med.* **2004**, *1*, 251–257. https://doi.org/10.1093/ecam/neh035.
97. Akhtar, N.M.; Naseer, R.; Farooqi, A.Z.; Aziz, W.; Nazir, M. Oral enzyme combination versus diclofenac in the treatment of osteoarthritis of the knee—A double-blind prospective randomized study. *Clin. Rheumatol.* **2004**, *23*, 410–415. https://doi.org/10.1007/s10067-004-0902-y.
98. Renzini, G.; Varengo, M. Absorption of tetracycline in presence of bromelain after oral administration. *Arzneim.-Forsch./Drug Res.* **1972**, *22*, 410–412.
99. Bradbrook, I.; Morrison, P.; Rogers, H. The effect of bromelain on the absorption of orally administered tetracycline. *Br. J. Clin. Pharmacol.* **1978**, *6*, 552–554. https://doi.org/10.1111/j.1365-2125.1978.tb00888.x.
100. Tinozzi, S.; Venegoni, A. Effect of bromelain on serum and tissue levels of amoxycillin. *Drugs Exp. Clin. Res.* **1978**, *4*, 39–44.
101. Tumilaar, S.G.; Siampa, J.P.; Fatimawali; Kepel, B.J.; Niode, N.J.; Idroes, R.; Rakib, A.; Emran, T.B.; Tallei, T.E. Potential of leaf extract of Pangium edule Reinw as HIV-1 protease inhibitor: A computational biology approach. *J. Appl. Pharm. Sci.* **2021**, *11*, 101–110. https://doi.org/10.7324/JAPS.2021.110112.
102. Batkin, S.; Taussig, S.J.; Szekerezes, J. Antimetastatic effect of bromelain with or without its proteolytic and anticoagulant activity. *J. Cancer Res. Clin. Oncol.* **1988**, *114*, 507–508. https://doi.org/10.1007/BF00391501.
103. Bristy, T.A.; Barua, N.; Tareq, A.M.; Sakib, S.A.; Etu, S.T.; Chowdhury, K.H.; Jyoti, M.A.; Aziz, M.; Ibn, A.; Reza, A. Deciphering the pharmacological properties of methanol extract of Psychotria calocarpa leaves by in vivo, in vitro and in silico approaches. *Pharmaceuticals* **2020**, *13*, 183. https://doi.org/10.3390/ph13080183.
104. Shukuru, W.; Kagira, J.; Maina, N. Toxicity, anthelmintic efficacy and proteolytic activity of chitosan-encapsulated bromelain within the gastrointestinal tract of small east african goats. *World’s Vet. J.* **2020**, *10*, 190–198. https://doi.org/10.36380/scil.2020.wvj25.
105. Rakesh, R.L.; Prasad, A.; Kumar, D.; Sankar, M.; Nasir, A.; Latchumikanthan, A.; Kushwaha, B. In vitro evaluation of anthelmintic efficacy of bromelain against goat gastrointestinal nematodes. *J. Vet. Parasitol.* **2016**, *30*, 68–74.
106. Saptarini, N.M.; Rahayu, D.; Kartikawati, E. Immunomodulatory Activity of Crude Bromelain of Pineapple (*Ananas comosus* (L.) Merr.) Crown from Subang District, Indonesia. *Res. J. Pharm. Technol.* **2020**, *13*, 5177–5182. https://doi.org/10.5958/0974-360X.2020.00905.1.
107. Golezar, S. Ananas comosus effect on perineal pain and wound healing after episiotomy: A randomized double-blind placebo-controlled clinical trial. *Iran. Red Crescent Med. J.* **2016**, *18*, e21019. https://doi.org/10.5812/ircmj.21019.
108. Majid, O.W.; Al-Mashhadani, B.A. Perioperative bromelain reduces pain and swelling and improves quality of life measures after mandibular third molar surgery: A randomized, double-blind, placebo-controlled clinical trial. *J. Oral Maxillofac. Surg.* **2014**, *72*, 1043–1048. https://doi.org/10.1016/j.joms.2013.12.035.
109. Walker, A.F.; Bundy, R.; Hicks, S.M.; Middleton, R.W. Bromelain reduces mild acute knee pain and improves well-being in a dose-dependent fashion in an open study of otherwise healthy adults. *Phytomedicine* **2002**, *9*, 681–686. https://doi.org/10.1078/094471102321621269.
110. Bakare, A.O.; Owoyele, B.V. Bromelain reversed electrolyte imbalance in the chronically constricted sciatic nerve of Wistar rats. *Naunyn. Schmiedebergs. Arch. Pharmacol.* **2020**, *393*, 457–467. https://doi.org/10.1007/s00210-019-01744-w.
111. Mallik, D.; Deb, L.; Gandhare, B.; Bhattacharjee, C. Evaluation of *Ananas comosus* Fruit for Antiulcer Potentials on Experimental Animals. *J. Harmon. Res. Appl. Sci.* **2019**, *7*, 89. https://doi.org/10.30876/johr.7.2.2019.89-97.
112. Rahman, J.; Tareq, A.M.; Hossain, M.M.; Sakib, S.A.; Islam, M.N.; Uddin, A.B.M.N.; Hoque, M.; Nasrin, M.S.; Ali, M.H.; Caiazzo, E.; et al. Biological evaluation, DFT calculations and molecular docking studies on the antidepressant and cytotoxicity activities of Cycas pectinata Buch.-Ham. Compounds. *Pharmaceuticals* **2020**, *13*, 232. https://doi.org/10.3390/ph13090232.
113. Tassman, G.C.; Zafran, J.N.; Zayon, G.M. A Double-Blind Crossover Study of a Plant Proteolytic Enzyme in Oral Surgery. *J. Dent. Med.* **1965**, *20*, 51–54.
114. Rakib, A.; Ahmed, S.; Islam, M.A.; Haye, A.; Uddin, S.N.; Uddin, M.M.N.; Hossain, M.K.; Paul, A.; Emran, T.B. Antipyretic and hepatoprotective potential of Tinospora crispa and investigation of possible lead compounds through in silico approaches. *Food Sci. Nutr.* **2020**, *8*, 547–556. https://doi.org/10.1002/fsn3.1339.
115. Secor, E.R., Jr.; Shah, S.J.; Guernsey, L.A.; Schramm, C.M.; Thrall, R.S. Bromelain Limits Airway Inflammation in an Ovalbumin-induced Murine Model of Established Asthma. *Altern. Ther. Health Med.* **2012**, *18*, 9–17.
116. Secor, E.R.; Carson, W.F., IV; Cloutier, M.M.; Guernsey, L.A.; Schramm, C.M.; Wu, C.A.; Thrall, R.S. Bromelain exerts anti-inflammatory effects in an ovalbumin-induced murine model of allergic airway disease. *Cell. Immunol.* **2005**, *237*, 68–75. https://doi.org/10.1016/j.cellimm.2005.10.002.
117. Jaber, R. Respiratory and allergic diseases: From upper respiratory tract infections to asthma. *Prim. Care Clin. Off. Pract.* **2002**, *29*, 231–261. https://doi.org/10.1016/S0095-4543(01)00008-2.
118. Secor, E.R.; Szczepanek, S.M.; Castater, C.A.; Adami, A.J.; Matson, A.P.; Rafti, E.T.; Guernsey, L.; Natarajan, P.; McNamara, J.T.; Schramm, C.M.; et al. Bromelain inhibits allergic sensitization and murine asthma via modulation of dendritic cells. *Evid. Based Complement. Altern. Med.* **2013**, *2013*, 702196. https://doi.org/10.1155/2013/702196.
119. Uddin, M.Z.; Rana, M.S.; Hossain, S.; Dutta, E.; Ferdous, S.; Dutta, M.; Emran, T.B. In vivo neuroprotective, antinociceptive, anti-inflammatory potential in Swiss albino mice and in vitro antioxidant and clot lysis activities of fractionated Holigarna longifolia Roxb. bark extract. *J. Complement. Integr. Med.* **2019**, *17*, 1–10. https://doi.org/10.1515/jcim-2019-0102.
120. Someshwar, U.S.S. Pityriasislichenoides. *Indian Pediatr.* **2012**, *49*, 936–941.
121. Shifah, F.; Tareq, A.M.; Sayeed, M.A.; Islam, M.N.; Emran, T.B.; Ullah, M.A.; Mukit, M.A.; Ullah, M. Antidiarrheal, cytotoxic and thrombolytic activities of methanolic extract of Hedychium coccineum leaves. *J. Adv. Biotechnol. Exp. Ther.* **2020**, *3*, 77–83. https://doi.org/10.5455/jabet.2020.d110.
122. Gabrielli, A.; Avvedimento, K.T. Scleroderma. *N. Engl. J. Med.* **2009**, *360*, 1989–2003.
123. Gaby, A.R. Natural remedies for scleroderma. *Altern. Med. Rev.* **2006**, *11*, 188–195.
124. Emran, T.B.; Rahman, M.A.; Uddin, M.M.N.; Dash, R.; Hossen, M.F.; Mohiuddin, M.; Alam, M.R. Molecular docking and inhibition studies on the interactions of Bacopa monnieri’s potent phytochemicals against Staphylococcus aureus. *DARU J. Pharma. Sci.* **2015**, *23*, 26. https://doi.org/10.1186/s40199-015-0106-9.
125. Hale, L.P.; Greer, P.K.; Sempowski, G.D. Bromelain treatment alters leukocyte expression of cell surface molecules involved in cellular adhesion and activation. *Clin. Immunol.* **2002**, *104*, 183–190. https://doi.org/10.1006/clim.2002.5254.
126. Hale, L.P.; Greer, P.K.; Trinh, C.T.; James, C.L. Proteinase activity and stability of natural bromelain preparations. *Int. Immunopharmacol.* **2005**, *5*, 783–793. https://doi.org/10.1016/j.intimp.2004.12.007.
127. Fahad, F.I.; Barua, N.; Islam, M.S.; Sayem, S.A.J.; Barua, K.; Uddin, M.J.; Chy, M.N.U.; Adnan, M.; Islam, M.N.; Sayeed, M.A.; et al. Investigation of the Pharmacological Properties of Lepidagathis hyaline Nees through Experimental Approaches. *Life* **2021**, *11*, 180. https://doi.org/10.3390/life11030180.
128. Praveen, N.C.; Rajesh, A.; Madan, M.; Chaurasia, V.R.; Hiremath, N.V.; Sharma, A.M. In vitro Evaluation of Antibacterial Efficacy of Pineapple Extract (Bromelain) on Periodontal Pathogens. *J. Int. Oral Health JIOH* **2014**, *6*, 96–98.
129. Giller, F.B. The effect of bromelain on levels of penicillin in the cerebrospinal fluid of rabbits. *Am. J. Pharm. Sci. Support. Public Health* **1962**, *134*, 238–244.
130. Lotz-Winter, H. On the pharmacology of bromelain: An update with special regard to animal studies on dose-dependent effects. *Planta Med.* **1990**, *56*, 249–253. https://doi.org/10.1055/s-2006-960949.
131. Bahde, R.; Palmes, D.; Minin, E.; Stratmann, U.; Diller, R.; Haier, J.; Spiegel, H.U. Bromelain Ameliorates Hepatic Microcirculation After Warm Ischemia. *J. Surg. Res.* **2007**, *139*, 88–96. https://doi.org/10.1016/j.jss.2006.10.004.
132. Guha, B.; Arman, M.; Islam, M.N.; Tareq, S.M.; Rahman, M.M.; Sakib, S.A.; Mutsuddy, R.; Tareq, A.M.; Emran, T.B.; Alqahtani, A.M. Unveiling pharmacological studies provide new insights on Mangifera longipes and Quercus gomeziana. *Saudi J. Biol. Sci.* **2021**, *28*, 183–190. https://doi.org/10.1016/j.sjbs.2020.09.037.
133. Tona, M.R.; Tareq, A.M.; Sayeed, M.A.; Mahmud, M.H.; Jahan, I.; Sakib, S.A.; Shima, M.; Emran, T.B. Phytochemical screening and in vitro pharmacological activities of methanolic leaves extract of Caryota mitis. *J. Adv. Biotechnol. Exp. Ther.* **2020**, *3*, 109–115. https://doi.org/10.5455/jabet.2020.d114.
134. Tareq, A.M.; Sohel, M.; Uddin, M.; Mahmud, M.H.; Hoque, M.; Reza, A.A.; Nasrin, M.S.; Kader, F.B.; Emran, T.B. Possible neuropharmacological effects of Apis cerana indica beehive in the Swiss Albino mice. *J. Adv. Biotechnol. Exp. Ther.* **2020**, *3*, 128–134. https://doi.org/10.5455/jabet.2020.d117.
135. Kargutkar, S.; Brijesh, S. Anti-rheumatic activity of *Ananas comosus* fruit peel extract in a complete Freund’s adjuvant rat model. *Pharm. Biol.* **2016**, *54*, 2616–2622. https://doi.org/10.3109/13880209.2016.1173066.
136. Hu, Y.; Wang, J.; Zhi, Z.; Jiang, T.; Wang, S. Facile synthesis of 3D cubic mesoporous silica microspheres with a controllable pore size and their application for improved delivery of a water-insoluble drug. *J. Colloid Interface Sci.* **2011**, *363*, 410–417. https://doi.org/10.1016/j.jcis.2011.07.022.
137. Parodi, A.; Haddix, S.G.; Taghipour, N.; Scaria, S.; Taraballi, F.; Cevenini, A.; Yazdi, I.K.; Corbo, C.; Palomba, R.; Khaled, S.Z.; et al. Bromelain surface modification increases the diffusion of silica nanoparticles in the tumor extracellular matrix. *ACS Nano* **2014**, *8*, 9874–9883. https://doi.org/10.1021/nn502807n.
138. Couto, C.; Vitorino, R.; Daniel-da-Silva, A.L. Gold nanoparticles and bioconjugation: A pathway for proteomic applications. *Crit. Rev. Biotechnol.* **2017**, *37*, 238–250. https://doi.org/10.3109/07388551.2016.1141392.
139. Khan, S.; Danish Rizvi, S.M.; Avaish, M.; Arshad, M.; Bagga, P.; Khan, M.S. A novel process for size controlled biosynthesis of gold nanoparticles using bromelain. *Mater. Lett.* **2015**, *159*, 373–376. https://doi.org/10.1016/j.matlet.2015.06.118.
140. Pereira De Sousa, I.; Cattoz, B.; Wilcox, M.D.; Griffiths, P.C.; Dalgliesh, R.; Rogers, S.; Bernkop-Schnürch, A. Nanoparticles decorated with proteolytic enzymes, a promising strategy to overcome the mucus barrier. *Eur. J. Pharm. Biopharm.* **2015**, *97*, 257–264. https://doi.org/10.1016/j.ejpb.2015.01.008.
141. Melinda Molnar, R.; Bodnar, M.; Hartmann, J.F.; Borbely, J. Preparation and characterization of poly(acrylic acid)-based nanoparticles. *Colloid Polym. Sci.* **2009**, *287*, 739–744. https://doi.org/10.1007/s00396-009-2033-0.
142. Nagpal, K.; Singh, S.K.; Mishra, D.N. Chitosan nanoparticles: A promising system in novel drug delivery. *Chem. Pharm. Bull.* **2010**, *58*, 1423–1430. https://doi.org/10.1248/cpb.58.1423.
143. Ataide, J.A.; Gérios, E.F.; Cefali, L.C.; Fernandes, A.R.; Teixeira, M.d.C.; Ferreira, N.R.; Tambourgi, E.B.; Jozala, A.F.; Chaud, M.V.; Oliveira-Nascimento, L.; et al. Effect of polysaccharide sources on the physicochemical properties of Bromelain-Chitosan nanoparticlesw. *Polymers* **2019**, *11*, 1681. https://doi.org/10.3390/polym11101681.
144. Pauzi, A.Z.M.; Yeap, S.K.; Abu, N.; Lim, K.L.; Omar, A.R.; Aziz, S.A.; Chow, A.L.T.; Subramani, T.; Tan, S.G.; Alitheen, N.B. Combination of cisplatin and bromelain exerts synergistic cytotoxic effects against breast cancer cell line MDA-MB-231 in vitro. *Chin. Med.* **2016**, *11*, 46. https://doi.org/10.1186/s13020-016-0118-5.
145. Kritis, P.; Karampela, I.; Kokoris, S.; Dalamaga, M. The combination of bromelain and curcumin as an immune-boosting nutraceutical in the prevention of severe COVID-19. *Metab. Open* **2020**, *8*, 100066. https://doi.org/10.1016/j.metop.2020.100066.
146. Akhter, J.; Queromes, G.; Pillai, K.; Kepenekian, V.; Badar, S.; Mekkawy, A.; Frobert, E.; Valle, S.; Morris, D.L. The combination of Bromelain and Acetylcysteine (BromAc) synergistically inactivates SARS-CoV-2. *Viruses* **2021**, *13*, 425. https://doi.org/10.3390/v13030425.
147. Pekas, E.J.; Shin, J.; Headid, R.J.; Son, W.M.; Layec, G.; Yadav, S.K.; Scott, S.D.; Park, S.Y. Combined anthocyanins and bromelain supplement improves endothelial function and skeletal muscle oxygenation status in adults: A double-blind placebo-controlled randomised crossover clinical trial. *Br. J. Nutr.* **2021**, *125*, 161–171. https://doi.org/10.1017/S0007114520002548.
148. Rahman, M.A.; Sultana, R.; Emran, T.B.; Islam, M.S.; Rahman, M.A.; Chakma, J.S.; Rashid, H.U.; Hasan, C.M.M. Effects of organic extracts of six Bangladeshi plants on in vitro thrombolysis and cytotoxicity. *BMC Complement. Altern. Med.* **2013**, *13*, 25. https://doi.org/10.1186/1472-6882-13-25.
149. Orsini, R.A. Bromelain. *Plast. Reconstr. Surg.* **2006**, *118*, 1640–1644. https://doi.org/10.1097/01.prs.0000242503.50548.ee.
150. Taussig, S.J.; Yokoyama, M.M.; Chinen, A.; Onari, K.; Yamakido, M. Bromelain: A proteolytic enzyme and its clinical application. A review. *Hiroshima J. Med. Sci.* **1975**, *24*, 185–193.
151. Seligman, B. Bromelain: An anti-inflammatory agent. *Angiology* **1962**, *13*, 508–510. https://doi.org/10.1177/000331976201301103.
152. Cohen, A.; Goldman, J. Bromelains Therapy in Rheumatoid Arthritis. *Pa. Med. J.* **1964**, *67*, 27–30.
153. Klein, G.; Kullich, W. Short-term treatment of painful osteoarthritis of the knee with oral enzymes. A randomised, double-blind study versus diclofenac. *Clin. Drug Investig.* **2000**, *19*, 15–23. https://doi.org/10.2165/00044011-200019010-00003.
154. Tilwe, G.H.; Beria, S.; Turakhia, N.H.; Daftary, G.V.; Schiess, W. Efficacy and Tolerability of Oral Enzyme Therapy as Compared to Diclofenac in Active Osteoarthrosis of Knee Joint: An Open Randomized Controlled Clinical Trial. *J. Assoc. Physicians India* **2001**, *49*, 617–621.
155. Al Mahmud, Z.; Emran, T.B.; Qais, N.; Bachar, S.C.; Sarker, M.; Uddin, M.M.N. Evaluation of analgesic, anti-inflammatory, thrombolytic and hepatoprotective activities of roots of *Premna esculenta* (Roxb). *J. Basic Clin. Physiol. Pharmacol.* **2016**, *27*, 63–70. https://doi.org/10.1515/jbcpp-2015-0056.
156. Ordesi, P.; Pisoni, L.; Nannei, P.; Macchi, M.; Borloni, R.; Siervo, S. Therapeutic efficacy of bromelain in impacted third molar surgery: A randomized controlled clinical study. *Quintessence Int.* **2014**, *45*, 679–684. https://doi.org/10.3290/j.qi.a32237.
157. Baur, X.; Fruhmann, G. Allergic reactions, including asthma, to the pineapple protease bromelain following occupational exposure. *Clin. Exp. Allergy* **1979**, *9*, 443–450. https://doi.org/10.1111/j.1365-2222.1979.tb02507.x.
158. Masson, M. Bromelain in blunt injuries of the locomotor system. A study of observed applications in general practice. *Fortschr. Med.* **1995**, *113*, 303–306.
159. Cirelli, M.G.; Smyth, R.D. Effects of bromelain anti-edema therapy on coagulation, bleeding, and prothrombin times. *J. New Drugs* **1963**, *3*, 37–39. https://doi.org/10.1002/j.1552-4604.1963.tb00060.x.
160. Rosenberg, L.; Shoham, Y.; Krieger, Y.; Rubin, G.; Sander, F.; Koller, J.; David, K.; Egosi, D.; Ahuja, R.; Singer, A.J. Minimally invasive burn care: A review of seven clinical studies of rapid and selective debridement using a bromelain-based debriding enzyme (Nexobrid^®^). *Ann. Burns Fire Disasters* **2015**, *28*, 264–274.

With this correction, the order of some references has been adjusted accordingly. The authors state that the scientific conclusions are unaffected. This correction was approved by the Academic Editor. The original publication has also been updated.
